# Species-level taxonomic characterization of gut microbiota in HIV-infected individuals

**DOI:** 10.3389/fmicb.2025.1657388

**Published:** 2025-08-29

**Authors:** Jiali Chen, Tingting Yuan, Han Zheng, Lianfeng Li, Ji Pu, Shan Lu, Yamin Sun, Wenchao Lin, Jun Chen, Mingquan Guo, Yubin Lu, Zhaoqin Zhu, Jing Yang, Jianguo Xu

**Affiliations:** ^1^School of Medicine, Research Institute of Public Health, Nankai University, Tianjin, China; ^2^National Key Laboratory of Intelligent Tracking and Forecasting for Infectious Diseases, National Institute for Communicable Disease Control and Prevention, Chinese Center for Disease Control and Prevention, Beijing, China; ^3^Shanghai Public Health Clinical Center, Fudan University, Shanghai, China; ^4^Institute of EcoHealth, School of Public Health, Cheeloo College of Medicine, Shandong University, Jinan, China; ^5^National Key Laboratory of Intelligent Tracking and Forecasting for Infectious Diseases, Beijing Ditan Hospital, Capital Medical University, Beijing, China; ^6^Uniteomics Tianjin Biotechnology Co., Ltd., Tianjin, China; ^7^Research Center for Reverse Microbial Etiology, Workstation of Academician, Shanxi Medical University, Taiyuan, China

**Keywords:** HIV, gut microbiota, enterotype, immune status, species level

## Abstract

Most current HIV gut microbiota studies, based on 16S rRNA gene sequencing, are limited to the genus level. Since different genera encompass several to hundreds of species, in this study, we performed research at the species level based on the HGMAD database. This cross-sectional study investigated differences in gut microbiota between healthy control (HC) subjects and people with HIV (PWH) at the species level, characterized the specific enterotype and the correlation between gut microbiota patterns and immune status in PWH. We recruited 114 individuals in the PWH group and 58 individuals in the HC group, from whom fecal samples were collected for 16S rRNA gene sequencing of the V3–V4 region. Significant differences in α diversity and β diversity (*p*-values < 0.05) were observed between the two groups. Compared to the HC group, the PWH group exhibited increased opportunistic pathogens and decreased commensal bacteria. Additionally, 114 species or higher taxa were enriched, while 49 species or higher taxa were depleted in the PWH group relative to those in the HC group. The gut microbiota of the HC group was categorized into enterotype *Bacteroides* (ET-B) and enterotype *Prevotella* (ET-P), whereas that of the PWH group exhibited ET-B, ET-P and enterotype *Enterobacter* (ET-E). ET-E was a unique enterotype in the PWH group, in which the abundance of *Enterobacter* was significantly increased. The absolute number of CD4^+^T lymphocytes and CD4/CD8 ratio were lower in ET-E HIV-infected individuals compared to those in the other two enterotypes. Ten signatures in three enterotypes showed high accuracy for distinguishing high and low CD4^+^T-cell counts groups, as well as high and low CD4/CD8 ratio groups, and the areas under curves were 0.831 (95%CI: 0.734–0.928) and 0.815 (95%CI: 0.721–0.909). *Spearman’s* correlation analysis revealed that five signatures enriched in the ET-E group were positively correlated with the absolute number of CD8^+^T lymphocytes. To sum up, compared to the HC group, the gut microbiota of PWH group exhibit reduced microbial diversity, an overall distinct microbial structure, and increased opportunistic pathogens. Furthermore, a novel enterotype (ET-E) was identified in HIV patients. Signatures based on different enterotypes can accurately and effectively discern the immune status of PWH, suggesting that the microbial composition of HIV infection is associated with the immune status.

## 1 Introduction

Acquired immune deficiency syndrome (AIDS) is an infectious disease caused by the human immunodeficiency virus (HIV). HIV infection is a global public health problem that has posed a major challenge. According to the HIV data reported by the World Health Organization (WHO), an estimated 39.9 million people were living with HIV at the end of 2023. In 2023, 630,000 people died from HIV-related causes globally. It has claimed an estimated 42.3 million lives so far (WHO, 2023). The human gut is a complex microbial ecosystem or microbiome and consists of 3.9 × 10^13^ bacteria (Sender et al., 2016). The gut microbiota is known to play key roles in many physiological processes, including immune response (Xiong et al., 2015). In addition to exploring behavioral aspects (Bien-Gund et al., 2022; Mlakar et al., 2023), the investigation of the role and mechanisms of gut microbiota in the development of HIV infection has also become a growing focus in academic research. The intestine is an important part of HIV infection, accumulating a large amount of HIV, which is the real virus reservoir of AIDS victims (Epeldegui et al., 2018; Mingjun et al., 2022). Regardless of the sites of HIV infection, the intestinal mucosa is the main site of early infection and replication. After contracting HIV, CD4^+^T lymphocytes in the intestinal mucosa of infected individuals are selectively depleted within 2 to 3 weeks (Brenchley et al., 2004). Furthermore, HIV infection not only disrupts the integrity of the intestinal epithelium but also damages the intestinal immune barrier. These results broke the balance of intestinal microbiota community structure, resulting in dysbiosis (Klatt et al., 2013). Compared to uninfected individuals, the gut microbiota diversity of PWH is altered. Generally, the gut microbiota diversity in PWH is reduced (Tuddenham et al., 2020).

However, most current gut microbiota studies in HIV infection, based on 16S rRNA gene sequencing, are limited to the genus level (Yarza et al., 2014). Different genera encompass several (e.g., *Escherichia* and *Shigella* including 6 and 4 species, respectively) or hundreds of species (e.g., *Streptococcus*) (Picker and Wing, 2016; Jabbour and Lartigue, 2021; Cobo-Simón et al., 2023). The physiological roles of different species within the same genus varies greatly. For instance, within the *Streptococcus* genus, *Streptococcus lactis* is probiotic bacteria, whereas *Streptococcus pneumoniae* is pathogenic bacteria (Krzyściak et al., 2013). The inferences drawn from the analysis results at the genus level may not be entirely accurate, hence the taxonomic diversity and composition of the gut microbiota should not be limited to the genus level. In this study, we performed the research at the species level. We further clarified the alterations of gut microbiota composition and diversity in people with HIV (PWH) at the species level. We also explored the relationship between differential species in enterotypes and immune status of PWH.

## 2 Materials and methods

### 2.1 Study population and sample collection

The PWH infected through sexual transmission who were undergoing blood and stool tests were recruited as the case group. Eligibility criteria for the PWH group included individuals (i) over 18 years of age, (ii) with a positive HIV antibody test, and (iii) without comorbidities such as hypertension, diabetes, or coronary heart disease, which may potentially affect the gut microbiota. Information was collected on the absolute number of CD4^+^T lymphocytes, the absolute number of CD8^+^T lymphocytes and CD4/CD8 ratio in peripheral blood. Based on previous studies, we divided participants into two immune status groups (CD4^+^ T-cell counts < 350 cells/μL and 350 cells/μL; CD4/CD8 ratio < 0.5 and ≥ 0.5) (Lu et al., 2015; Chandiwana et al., 2023). The healthy control (HC) group do not have sexually transmitted infections. Healthy group contained 58 healthy individuals which came from our previous project (Yang et al., 2020). To recruit subjects, we had established inclusion and exclusion criteria to determine “healthy” individuals, mainly consist of individuals aged between 18 and 60, whose physical examination indicators are within the normal range, and who have no metabolic, mental or infectious diseases. The remaining fecal samples from these participants were collected for our study after testing at the hospital’s laboratory department.

### 2.2 16S rRNA gene amplification and sequencing

DNA extraction from fecal samples was performed using QlAamp Fast DNA Stool Mini Kit (Qiagen, Germany) following the manufacturer’s instructions. The V3-V4 region of the 16S rRNA gene was amplified from the extracted DNA using barcodes primers of 338F (5’-ACTCCTACGGGAGGCAGCAG-3′) and 806R (5’-GGACTACHVGGGTWTCTAAT-3′). The quality inspection was conducted on all samples to meet the sequencing requirements. Finally, all qualified samples were sequenced by Shanghai Major Bio-pharm Biotechnology Co., Ltd.

### 2.3 Overview of data processing and analysis

#### 2.3.1 Bioinformatics analysis

The raw data were first subjected to quality control and processed using fastp (Chen et al., 2018) to get high-quality reads. Then DADA2 (Divisive Amplicon Denoising Algorithm) in QIIME2 was used to generated amplicon sequence variants (ASVs) (Bolyen et al., 2019). Taxonomic classification of these ASVs was conducted using the VSEARCH classifier against the HGMAD (Human Gut Microbiome Analysis Database, DOI: 10.6084/m9.figshare.27281403) (Wang et al., 2025). The species-level characterization in this study mainly relies on the HGMAD which is constructed by collecting 16S rRNA gene reference sequences of selected species and subspecies from two authoritative databases including LPSN (List of Prokaryotic names with Standing in Nomenclature) and the NCBI (National Center for Biotechnology Information) Ref NR database. To expand the database, we incorporated candidate sequences from SILVA and added sequencing data from 120 healthy individuals (Yang et al., 2020). From this nearly full length 16S rRNA gene reference sequences dataset, a non-redundant database for the V3–V4 regions (positions 341–806) was extracted. Finally, using this incorporated database, we identified flexible classification thresholds for 674 families, 3,661 genera, and 15,735 species, enabling more precise species-level identification. For more details, you can refer to our published article (Wang et al., 2025). ASVs could be assigned to known species and potential new species at the species level, or higher taxa at the family or higher taxonomic levels. Additionally, known species were categorized into probiotic, commensal, and potential pathogenic bacteria according to the previous literature (Yang et al., 2020; Bartlett et al., 2022) and the Probio database (a database of probiotics functions and linenges, https://bidd.group/probio/homepage.htm). Microbial community function prediction was performed by PICRUSt2 (Phylogenetic Investigation of Communities by Reconstruction of Unobserved States) (Douglas et al., 2020) on the basis of the Kyoto Encyclopedia of Genes and Genomes (KEGG) database.

#### 2.3.2 Analysis of the relative prevalence for ASVs

Relative abundance of a given ASV in each sample was normalized as “(ASV reads/total reads) × 100 per sample.” The prevalence of a given ASV in each sample was determined by “(the count of an ASV across all samples/the total number of all samples) × 100 per sample.” According to the prevalence less than 10, 10 to 60% and greater than 60% of the population, all ASVs were classified into three groups of low, medium and high prevalent bacteria groups, respectively. The population with the prevalence less than 10% was considered allochthonous and temporary inhabitants of the gut microbial environment (McNulty et al., 2011).

#### 2.3.3 Classification of the gut microbiota based on enterotypes

Gut enterotypes were classified based on the method described previously (Arumugam et al., 2011). The Jensen-Shannon distance (JSD) according to the abundance of gut microbiota at the genus level was calculated and the partitioning around medoids (PAM) algorithm clustering was performed. The Calinski-Harabasz (CH) index was applied to estimate the optimal number of clusters, as it serves as an internal validation metric for evaluating clustering quality. Its fundamental principle assesses clustering performance by quantifying the ratio of between-cluster dispersion to within-cluster dispersion. CH index has become the metric of choice for enterotype analysis in microbiota studies, particularly for determining the optimal number of enterotypes, owing to its computational tractability, statistical robustness, and precise evaluation of spherical clusters (Arumugam et al., 2011). The silhouette coefficient was used to assess the statistical significance.

#### 2.3.4 Statistical analysis

Alpha diversity metrics, including Chao1 and Shannon indices, were calculated using the “vegan” package in R software v4.3.1^1^ and the inter-group differences were analyzed by Wilcoxon rank-sum test. Principal coordinate analysis (PCoA) based on Bray-Curtis distance and permutational multivariate analysis of variance (PERMANOVA) using the adonis function were performed to assess beta diversity. Features were filtered by setting a minimum occurrence frequency (the proportion of samples with non-zero abundance) of 0.2, removing low-abundance bacteria. Differential bacterial taxa between the HC and PWH groups were identified using the “DESeq2” package, with the criteria of |log2FoldChange| > 1 and a false discovery rate (FDR) < 0.05. Significant differences in relative abundance of KEGG pathways between the HC and PWH groups were assessed using Wilcoxon rank-sum test with Benjamini–Hochberg (BH) FDR correction.

Chi-square or Fisher’s exact test was used to assess whether enterotypes were significant difference in different immune status of PWH. A two-step method was adopted to select signatures for different enterotypes. Initially, a random forest model (ntree = 500) identified the 10 most predominant species as candidates for distinguishing different enterotypes. Subsequently, linear discriminant analysis effect size (LEfSe) analysis was applied to the same dataset to detect species with significant differences among the three groups, with a threshold for LDA score > 2 and *p* < 0.05 (Gao et al., 2024). Finally, overlapping species from both methods were designated as signatures. Multivariate logistic regression analysis was conducted to evaluate the predictive ability of signatures for different immune status in PWH. The predictive probability values were used to plot the receiver operating characteristic (ROC) curve and calculate the area under ROC curve (AUC) for accuracy assessment. *Spearman’s* correlation analysis was used to examined correlations between signatures and immune status indicators.

## 3 Results

### 3.1 Study subjects

In our cross-sectional study, a total of 58 healthy individuals (HC) without sexual transmission and 114 HIV infected individuals (PWH) with sexual transmission were enrolled, and their fecal samples were collected. The characteristics of these participants were summarized in Table 1. In the HC group, the median age was 33.0 years, and 48% of the participants were male. In the PWH group, the median age was 43.5 years and 86% of the participants were male. The median CD4^+^T-cell count, CD8^+^T-cell count and CD4/CD8 ratio were 101.5 cells/μL, 562 cells/μL and 0.17, respectively. No information was available on whether the PWH had received antiretroviral therapy.

**Table 1:** Baseline characteristics of subjects in the healthy control (HC) and people with HIV (PWH) groups.

Characteristics	HC group	PWH group	*P*-value
Number of subjects	58	114	–
Gender male/female	28/30	98/16	<0.001
Age (years, IQR)	33.0 (26.5, 45.5)	43.5 (32.0, 55.0)	<0.001
CD4 + T-cell count (cells/μL, IQR)	–	101.5 (30.5, 296.0)	–
CD8 + T-cell count (cells/μL, IQR)	–	562.0 (359.3, 847.0)	–
CD4+/CD8 + T-cell ratio (IQR)	–	0.17 (0.07, 0.44)	–

Continuous variables are described with median and interquartile range, denoted as median (IQR). “–” indicates that the measurement was not performed.

### 3.2 Hierarchic taxonomic composition and classification of gut microbial community

**Figure 1:**
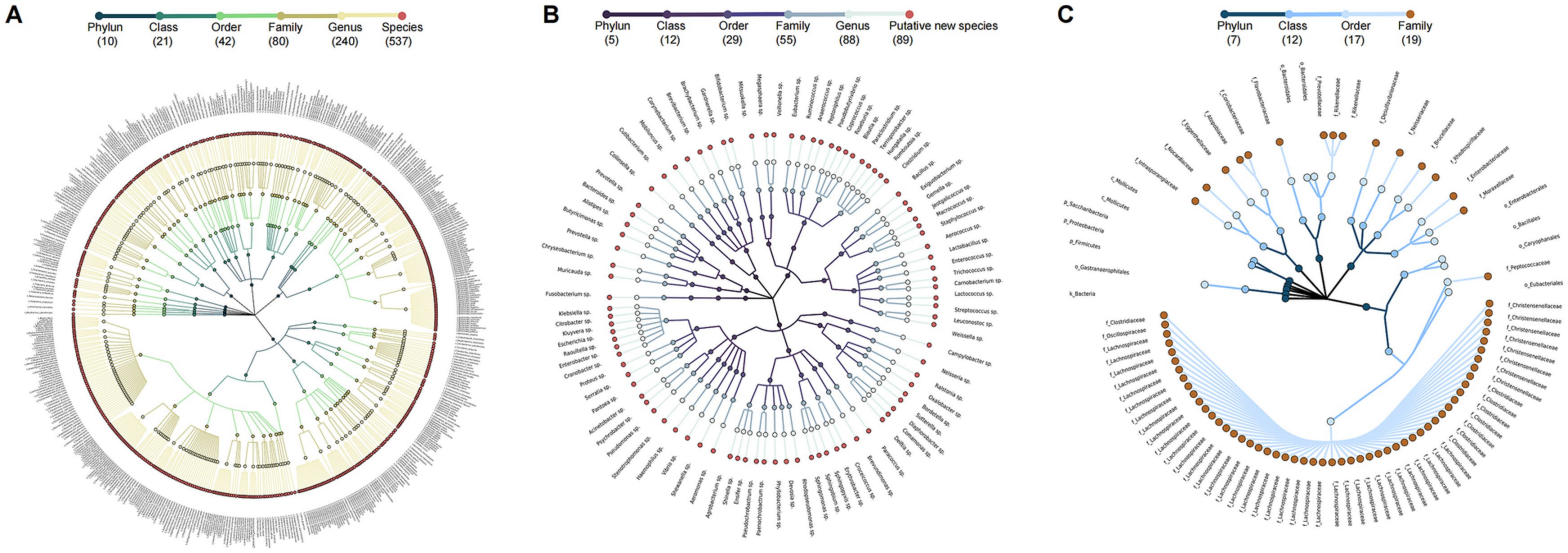
Taxonomic profiles of 708 ASV in the gut microbial community of PWH. A taxonomic tree of **(A)** 537 known species, **(B)** 89 potentially new species, and **(C)** 82 potentially higher taxa in PWH. Each dot represents an ASV. The descending hierarchical levels are expressed from the inner to the outer rings. The total number of ASVs at different hierarchical levels is displayed in brackets.

After quality filtering and chimera removal, 58 healthy individual samples in HC group yielded 5,082,998 high-quality reads, with an average of 36,888 reads per sample. Meanwhile, 114 PWH samples generated 10,782,734 high-quality reads, with an average 82,244 reads per sample. Denoising of the 16S rRNA gene reads yielded 635 ASVs and 708 ASVs for the HC and PWH groups, respectively. For the HC group, the 635 ASVs consisted of 454 known species, affiliated with 12 phyla, 23 classes, 47 orders, 79 families, and 214 genera, covering 81.97% of the total reads, as well as 81 potentially new species and 100 potentially higher taxa (Supplementary Table 1). The 708 ASVs were taxonomically classified into 537 known species, affiliated with 10 phyla, 21 classes, 42 orders, 80 families, and 240 genera, accounting for 58.06% of the total reads, along with 89 potentially new species and 82 potentially higher taxa in PWH group (Figure 1). The 82 potentially higher taxa comprised 1 ASV at the kingdom level, 3 ASVs at the phylum level, 2 ASVs at the class level, 7 ASVs at the order level and 69 ASVs at the family level.

### 3.3 PWH group with medium and high prevalent groups, while HC group with high prevalent bacteria group

Among the 708 ASVs in the PWH group, 470, 220, and 18 ASVs were classified into low, medium, and high prevalent bacteria groups, representing 4.00, 45.80, and 50.20% of the total reads, respectively (Supplementary Figure 1A). Similarly, of 635 ASVs in the HC group, 236, 218, and 181 ASVs were classified into low, medium, and high prevalent bacteria groups, accounting for 0.11, 1.84, and 98.05% of the total reads, respectively (Supplementary Figure 1B). Notably, the proportion of reads in the low- and mid-prevalence groups in people with HIV was higher than that in healthy control individuals, while the proportion of reads in the high-prevalence group was lower.

### 3.4 Reduced diversity of HIV gut microbiota compared to healthy individuals

In the PWH group, the Chao1 (Supplementary Figure 2A) and Shannon (Supplementary Figure 2B) indices showed a decreasing trend compared to the HC group (*p*-values< 0.05). PCoA analysis revealed a significant difference in gut microbial community structures between the two groups (*p* < 0.001) (Supplementary Figure 2C). To better understand the mechanisms of action of gut microbiota on the host, 537 known species in the PWH group and 454 known species in the HC group were classified into probiotic, commensal, and potentially pathogenic bacteria according to literature publication. In the HC group, there were 175 opportunistic pathogens, 242 commensal bacteria, and 37 probiotics, accounting for 13.66, 78.14, and 8.20% of the total reads, respectively. In the PWH group, there were 226 opportunistic pathogens, 265 commensal bacteria, and 46 probiotics, representing 26.82, 66.89, and 6.29% of the total reads, respectively (Supplementary Figure 3 and Supplementary Table 2). The most common opportunistic pathogens in the PHW group included *Ruminococcus gnavus*, *Parabacteroides distasonis*, *Veillonella parvula*, detected in 71.05, 63.16, and 74.56% of the samples, respectively.

**Figure 2:**
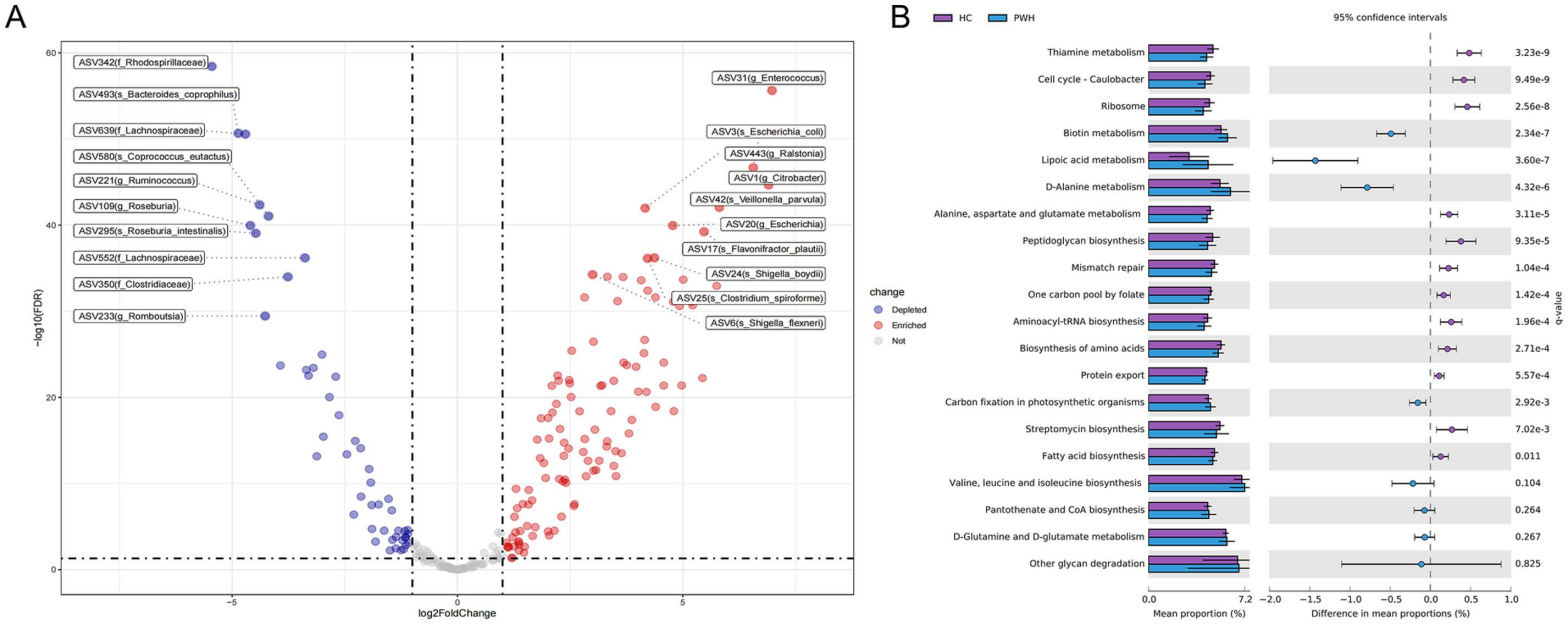
Statistically different bacterial between the PWH and HC groups. **(A)** Each point represents a species or higher taxon. The red points represent the significantly enriched species or higher taxa, the blue points represent the significantly depleted species or higher taxa, and the gray points represent the species or higher taxon with no significant difference between the two groups. The labels display the top ten enriched or depleted bacteria, respectively. **(B)** The relative abundance of the top 20 metabolic pathways based on level 3 KEGG Ortholog (KO) functional predictions were compared between the HC and PWH groups, with significance determined at an adjusted *p*-value (*q*-value) threshold of less than 0.05.

After filtering out low-abundance bacteria, the “DESeq2” package was used to identify differential bacteria between the HC and PWH groups. Compared to the HC group, the PWH group showed an increase of 114 ASVs, primarily including ASV17 (*Flavonifractor plautii*), ASV42 (*Veillonella parvula*), ASV25 (*Clostridium spiroforme*). Moreover, a total of 49 ASVs was decreased in the PWH group, mainly comprising ASV493 (*Bacteroides coprophilus*), ASV580 (*Coprococcus eutactus*), ASV295 (*Roseburia intestinalis*) and ASV480 (*Dialister hominis*) (Figure 2A and Supplementary Table 3). To predict and compare gut microbiota functions between the HC and PWH groups, this study employed PICRUSt2 software for microbiota function prediction based on the KEGG database and analyzed the prediction results using STAMP software. Among the top 20 KEGG third-level pathways, 16 pathways such as ‘thiamine metabolism’, ‘cell cycle-caulobacter’, ‘ribosome’ showed significant differences between the HC and PWH groups. Conversely, the remaining four pathways, namely ‘valine, leucine and isoleucine biosynthesis,’ ‘pantothenate and CoA biosynthesis,’ ‘d-glutamine and d-glutamate metabolism,’ and ‘other glycan degradation,’ exhibited no significant differences between the two groups (Figure 2B).

### 3.5 Enterotype E: a specific enterotype in PWH

**Figure 3:**
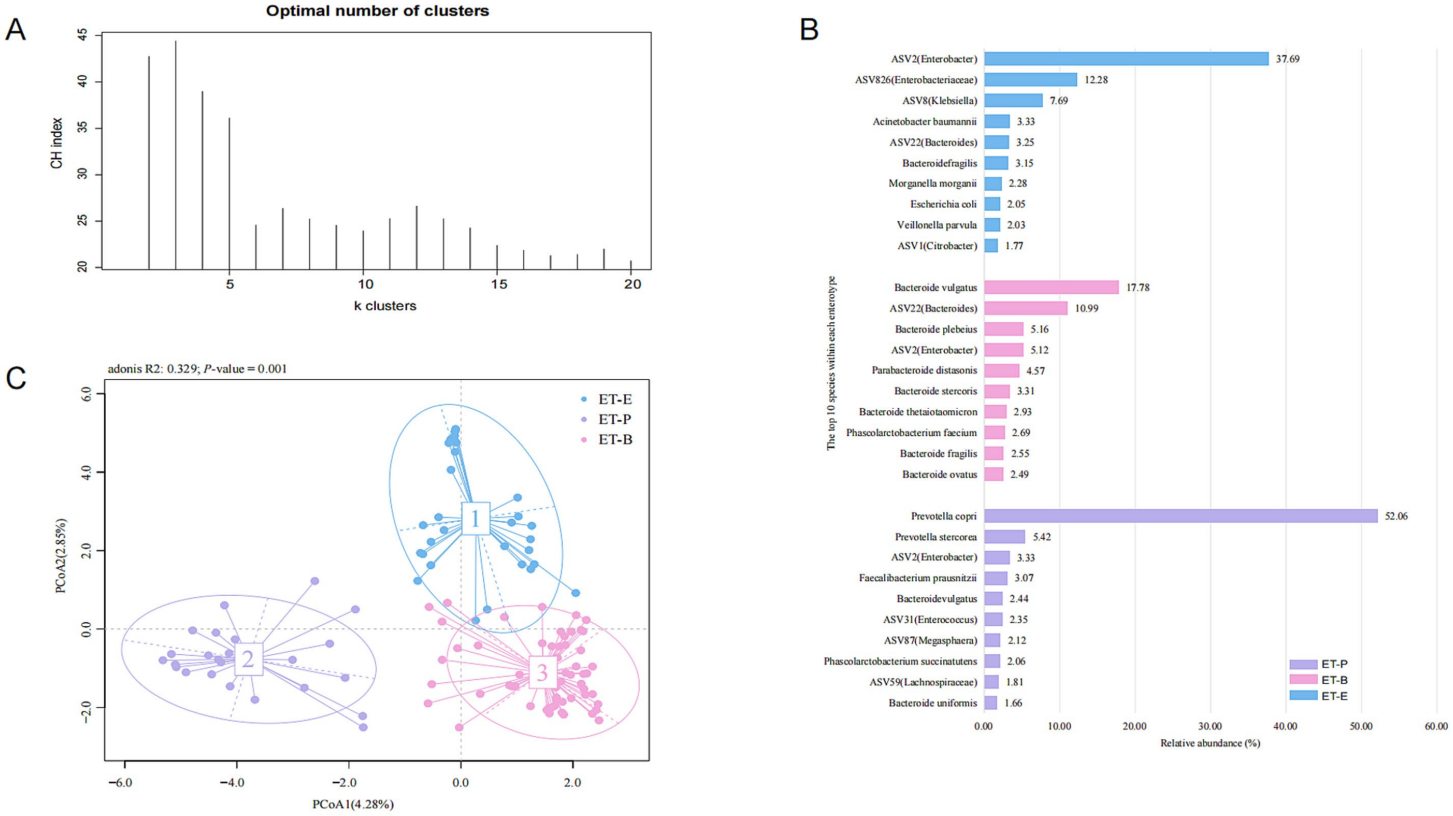
Enterotypes in the PWH group. **(A)** According to the maximum Calinski-Harabasz (CH) index, the optimal number of enterotypes of the PWH group were three clusters. **(B)** The principal co-ordinate analysis (PCoA) blot. **(C)** The relative abundance of the top ten species in the three enterotypes in the PWH group.

The gut microbiota of 58 healthy individuals was clustered into two enterotypes, dominated by *Bacteroides* (ET-B) and *Prevotella* (ET-P) (Supplementary Figure 4). The gut microbiota of 114 PWH was clustered into three enterotypes (Figure 3), with each enterotype dominated by *Bacteroides* (ET-B), *Prevotella* (ET-P) and *Enterobacter* (ET-E), respectively (Supplementary Figure 5). The clinical information among three enterotypes was detailed in Table 2. The silhouette index was 0.23. Alpha diversity analysis showed different richness and evenness among the three enterotypes (Supplementary Figure 6). Specifically, in the PWH group, the Chao1 index for ET-E was significantly lower than for the other two enterotypes.

**Table 2:** Baseline characteristics of three enterotypes HIV-infected subjects.

Characteristics	Enterotype B	Enterotype P	Enterotype E
Number of subjects	59	25	30
Gender male/female	49/10	23/2	26/4
Age (years)	45 (31,58)	37 (31.5, 48.5)	44 (31.75, 57)
CD4^+^ T-cell count (cells/mm3)	78.0 (23.0, 307.0)	174.0 (48.0, 521.0)	103.5 (45.8, 202.8)
CD4^+^ T-cell count < 350	81.63%	58.82%	100%
CD4^+^ T-cell count ≥ 350	18.37%	41.18%	0
CD8^+^ T-cell count (cells/mm3)	529.0 (314.5, 846.5)	513.0 (359.5, 791.5)	667.5 (479.5, 1214.0)
CD4^+^/CD8^+^ T-cell ratio	0.15 (0.06, 0.46)	0.23 (0.11, 0.92)	0.16 (0.09, 0.24)
CD4/CD8 ratio < 0.5	77.55%	52.94%	95.45%
CD4/CD8 ratio ≥ 0.5	22.45%	47.06%	4.55%

Continuous variables are described with median and interquartile range, denoted as median (IQR).

### 3.6 Enterotypes signatures are related to different immune status in people with HIV

**Figure 4:**
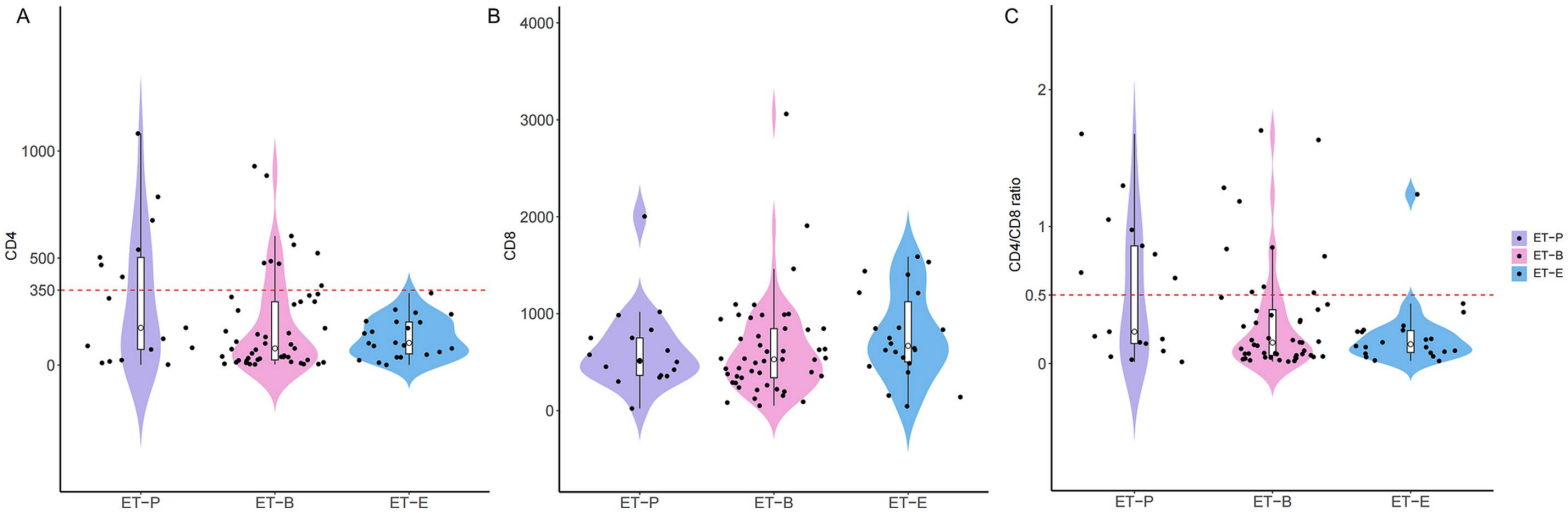
The distribution of T cell counts among three enterotypes of PWH. **(A)** CD4^+^T cell counts, **(B)** CD8^+^T cell counts and **(C)** CD4/CD8 ratio.

The distribution of CD4^+^T-cell counts, CD8^+^T-cell counts and CD4/CD8 ratio in three enterotypes of PWH were shown in Figure 4. Box-violin plots indicated that the density distribution of CD4^+^T-cell counts and CD4/CD8 ratio in the ET-E group differed from other enterotypes. The box-violin plot showed that all individuals in ET-E group had CD4^+^T-cell counts less than 350 cells/μL. Fisher’s exact tests revealed significant differences in enterotypes distribution between low (<350 cells/μL) and high (≥350 cells/μL) CD4^+^T-cell counts groups, as well as between low (<0.5) and high (≥ 0.5) CD4/CD8 ratio groups, with *p-*values of 0.003 and 0.007, respectively (Supplementary Figure 7).

**Figure 5:**
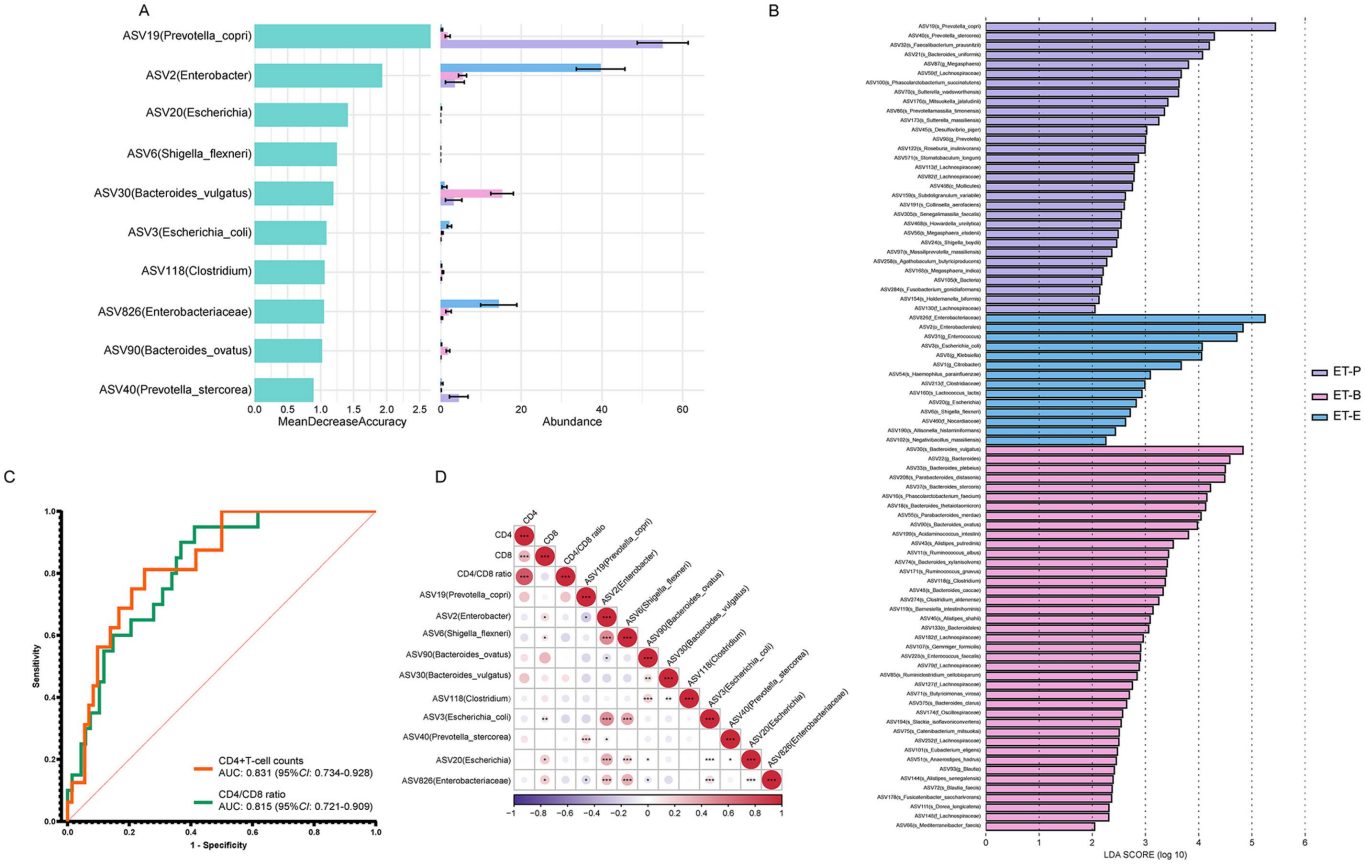
Identification of signatures and prediction, and *Spearman’s* correlation between the relative abundance of differential bacteria among different enterotypes of PWH and immune status indicators. **(A)** The random forest importance of top ten species and their relative abundance in three enterotypes. **(B)** Linear discriminant analysis (LDA) effect size analysis was used to distinguish three enterotypes. At the species level, LDA scores showed significant differences in microbiota composition among ET-P (purple), ET-B (pink), and ET-E (blue) groups. Only taxa with an LDA threshold > 2 were shown. **(C)** Prediction by ten signatures in three enterotypes. The area under the receiver operating characteristic (ROC) curve (AUC), with 95% confidence interval (CI). **(D)** *Spearman’s* correlation between the relative abundance of differential bacteria among different enterotypes of PWH and CD4^+^T-cell count, CD8^+^T-cell count, CD4/CD8 ratio. The purple gradient indicates negative correlation (−1–0), while the red gradient indicates positive correlation (0–1). *p < 0.05, **p < 0.01, ***p < 0.001.

In the follow-up analysis, a two-step method combining LEfSe analysis and random forest identified the top 10 ASVs, which were annotated ASV19 (*Prevotella copri*), ASV2 (*Enterobacter*), ASV6 (*Shigella flexneri*), ASV90 (*Bacteroides ovatus*), ASV30 (*Bacteroides vulgatus*), ASV118 (*Clostridium*), ASV3 (*Escherichia coli*), ASV40 (*Prevotella stercorea*), ASV20 (*Escherichia*), ASV826 (*Enterobacteriaceae*) among the three enterotypes (Figures 5A,B). Among these, ASV2 (*Enterobacter*), ASV6 (*S. flexneri*), ASV3 (*E. coli*), ASV20 (*Escherichia*), ASV826 (*Enterobacteriaceae*) were mainly enriched in the ET-E group. ASV19 (*P. copri*) and ASV40 (*P. stercorea*) were mainly enriched in the ET-P group, while ASV90 (*B. ovatus*), ASV30 (*B. vulgatus*) and ASV118 (*Clostridium*) were mainly enriched in the ET-B group. As shown in Figure 5C, these top 10 ASVs served as gut microbial signatures, effectively distinguished between low and high CD4^+^T-cell counts groups (AUC: 0.831, 95%CI: 0.734–0.928), and between low and high CD4/CD8 ratios groups (AUC: 0.815, 95%CI: 0.721–0.909).

Among the top 10 bacteria, ASV2 (*Enterobacter*), ASV20 (*Escherichia*), ASV826 (*Enterobacteriaceae*), ASV6 (*S. flexneri*) and ASV3 (*E. coli*), five signatures belonged to *Enterobacterales* were enriched in the ET-E group. *Spearman’s* correlation analysis revealed that these five signatures were positively correlated with CD8^+^T-cell counts (Figure 5D). Additionally, ASV19 (*P. copri*) exhibited a negative correlation with the relative abundance of ASV2 (*Enterobacter*) and ASV826 (*Enterobacteriaceae*). ASV90 (*B. ovatus*) showed a negative correlation with the relative abundance of ASV826 (*Enterobacteriaceae*) and ASV20 (*Escherichia*).

## 4 Discussion

In recent years, many domestic and foreign scholar teams have studied the relationship between gut microbiota and metabolic diseases, and even their association with mental disorders. However, there are relatively few reports on the microbiota of patients with infectious diseases. In this study, we attempted to collect fecal samples from HIV patients at a designated infectious disease hospital in China. Although using common V3-V4 amplicon sequencing, due to the use of a suited database, the microbiota could basically be annotated to the bacterial species level.

This study further confirms that HIV infection is associated with reduced gut microbiota diversity, and changes in its composition and structure. These findings are basically consistent with most prior research (Vujkovic-Cvijin et al., 2020; Boukadida et al., 2024; Tian et al., 2024; Zhou et al., 2024). Mingjun et al. (2022) reported the top five genera in HIV group as *Bacteroides*, *Prevotella*, *Fusobacterium*, *Lachnospira* and *Escherichia-Shigella*. In our study, the enriched gut microbiota of PWH included *Enterococcus*, *Escherichia-Shigella*, *Citrobacter*. Detailed classification of known species into probiotic, commensal and pathogenic bacteria revealed that compared to the HC group, the PWH group exhibited an increased and more fluctuant proportion of opportunistic pathogens, along with reduced proportions of commensal and probiotic bacteria. This indicates that HIV infection disrupts gut microbiota composition and function, a phenomenon termed “dysbiosis,” which is typically characterized by decreased commensal bacteria and increased potentially pathogenic bacteria (Dillon et al., 2016; Geng et al., 2020).

*Enterococcus* genus, comprising over 60 Gram-positive species, belongs to normal inhabitants in both human and animal. *Enterococci* have been historically used as probiotics in humans and slaughter animals (Franz et al., 2011). However, under certain conditions, they can pose latent risks and are thus considered as “pathobionts,” which has caused widespread concern (Xu et al., 2024). Lu et al. (2019) found that the amounts of *E. coli*, *Enterococcus faecalis*, and *Enterococcus faecium* were positively correlated with the content of TNF-α and IL-6, and *Enterococcus* was negatively correlated with CD4^+^T lymphocyte counts in AIDS/HIV patients. In addition, in the vitro study have shown that commensal *Enterococcus* can increases the activation and expansion of resident *E. coli*-reactive T cells resulting in increased HIV-1 replication and infection of IFN-γ and IL-17-producing CD4 T cells (Dillon et al., 2012). *Coprococcus* genus, generally considered beneficial, has been reported to decrease in abundance across a variety of disease states, such as depression (Yu et al., 2017), acute myeloid leukemia (Pötgens et al., 2024), and constipated obese children (Zhu et al., 2014). However, changes in this genus in HIV-infected individuals have not been reported. Only one shotgun metagenomics sequencing study reported a decrease of *Coprococcus comes* in anal swab samples in HIV-infected individuals (Lacunza et al., 2023). Based on 16S rRNA gene sequencing, there have been no specific studies on gut microbiota of PWH at the species level. In our study, we focused on the species level and found that the relative abundance of *C. eutactus* was lower in the PWH group compared to the HC group. *C. eutactus* is a short-chain fatty acid-producing bacterium, primarily generating acetic acid. Gavage with *C. eutactus* can reduce the concentrations of proinflammatory cytokines TNF-α, IL-1β, and IL-6, and increase anti-inflammatory factors, IL-4, IL-5, and IL-10 (Yang et al., 2023). The beneficial effects of beneficial bacteria similar to *C. eutactus* in HIV infection deserve further exploration. Overall, it can be seen that the altered gut microbiota in PWH group may play different roles in disease progression.

Enterotypes provide an in-depth understanding of gut microbiota characteristics and facilitate the gradual standardization of gut microbiota analysis, enhancing comprehension of human health and diseases status. The classification based on compositional patterns would potentiate microbiota-based diagnosis, treatment or disease prevention, with implications for personalized treatment through nutritional, microbial, and pharmaceutical interventions (Costea et al., 2018). Enterotype classification should ideally be based on samples of the same type (e.g., individuals with the same disease or in the same physiological state) to minimize the impact of significant confounding factors. Previous studies on the construction of enterotype in healthy individuals, patients, or animals have shown preferred community states emerging more clearly when no external factors influence the microbiota. The enterotype concept was first proposed in 2011, and three enterotypes were classified based on dominated genera: ET-B, ET-P, and enterotype *Ruminococcus* (ET-R) (Arumugam et al., 2011). Recent studies have explored enterotypes in various diseases, including HIV (Belda et al., 2024; Tito et al., 2024; Wu et al., 2024). Belda et al. (2024) found three major enterotypes (ET-B, ET-P, and ET-R) in HIV-infected individuals based on the dirichlet multinomial mixture (DMM) method of Holmes et al. (2012). Noguera-Julian et al. (2016) reported two enterotypes (ET-B and ET-P) in HIV-1-infected subjects. In our study, in addition to ET-B and ET-P, a novel enterotype (ET-E) characterized by the increased abundance of *Enterobacter* was clustered.

The characteristics of gut microbiota among three enterotypes were conducted to assess the correlation between immune status and gut microbiota in PWH. Our study revealed a significant association between enterotypes and immune status in PWH, especially PWH with ET-E showing a lower immune status. This finding suggests that HIV infection affects the gut microbiota homeostasis and prompts the transition of enterotypes from common ET-B and ET-P to ET-E. Studies indicate that the immune system impairment in PWH, particularly the depletion of CD4^+^T cells, leads to the loss of intestinal mucosal barrier function. Consequently, there is an increased translocation of gut microbiota and their metabolites across the mucosal barrier into the bloodstream, a process known as microbial translocation. This process is recognized as an important contributor to chronic immune activation (Brenchley et al., 2006; Marchetti et al., 2013). Immune system impairment, disruption of intestinal mucosal barrier function, and chronic inflammatory may be key factors that lead to the transition of the intestinal microbial community structure and thus trigger transition in enterotypes.

At the species level, this study revealed that *S. flexneri* and *E. coli* were positively correlated with CD8^+^T-cell counts, suggesting that gut microbiota play an important role in immune regulation in HIV-infected individuals. *S. flexneri*, a known pathogen, can cause intestinal inflammation (Schnupf and Sansonetti, 2019; Han et al., 2024). The key for *Shigella* pathogenicity lies in the multifaceted regulation of host signaling pathways via its type three secretion system (T3SS). The regulation includes the subversion of host innate immune defenses and the promotion of intracellular bacterial survival and dissemination, which leads to the initiation of a pro-inflammatory milieu (Brunner et al., 2019). *E. coli*, a commensal bacterium, can also encompasses pathogenic strains that can induce inflammation (Kittana et al., 2018). Its lipopolysaccharide (LPS) exhibits stronger immunogenic potential than that of other *Enterobacteria* (Garvey, 2024). Additionally, exposure to commensal *E. coli* alters HIV-1-induced mucosal CD4^+^T cell death pathways *ex vivo*, with potential consequences for mucosal inflammation, viral dissemination and systemic immune activation (Steele et al., 2014). Our species-level analysis of the gut microbiota further supports the viewpoint that specific bacterial species may play a potential role in immune activation and inflammation. However, this activation may culminate in T cell exhaustion, potentially accelerating HIV disease progression.

There are some limitations in our study. Firstly, our study was based on a limited number of Chinese subjects and did not include individuals from other countries. Due to ethical consideration and privacy concern, the specific information on the exact sexual behavior of patients was not collected in this study. Furthermore, the impact of antiretroviral therapy on the gut microbiota of PWH was not investigated. Additionally, the inclusion/exclusion criteria and methods used to find the main bacteria between the HC and PWH groups were not entirely consistent, which may lead to the inconsistency between our results and previous research. The prediction results of PICRUSt2 based on the taxonomy of 16S rRNA genes were for reference only. Despite these limitations, our study’s strength lies in its species-level analysis of gut microbiota. We hope future research can address these limitations to better understand the relationship between gut microbiota and PWH.

In summary, our study shows that, although the gut microbiota of people with HIV has decreased diversity, it still exhibits great variability in composition compared to healthy individuals. The identification of a novel HIV-associated enterotype has provided fresh insight on enterotype research. The relative abundance of *Escherichia-Shigella* correlated positively with CD8^+^T-cell counts, suggesting the potential role in immune activation and inflammation. These findings further supports that specific gut bacteria may contribute to immune activation and inflammation in HIV infection. Species-level analysis of gut microbiota could enhance our understanding of the link between gut microbiota and disease, paving the way for precision medicine based on gut microbiota.

## Data Availability

The original contributions presented in the study are publicly available. This data can be found here: Microbiology Data Center (NMDC), accession number NMDC10019425 (https://nmdc.cn/resource/genomics/project/detail/NMDC10019425).

## Footnotes

^1^ http://www.r-project.org/

## Supplementary material

SUPPLEMENTARY FIGURE 1 Low (<10%), medium (10–60%), and high (>60%) prevalent bacteria groups in PWH and HC. The numbers (left axis) and the relative abundance (right axis) of the ASVs in individuals with the 5% interval. Panel **(A)** in the PWH group. Panel **(B)** in the HC group.

SUPPLEMENTARY FIGURE 2 Alpha diversity and beta diversity between PWH and HC groups. **(A)** Chao1 index, **(B)** Shannon index, **(C)** β diversity. *P*-values < 0.001. Boxes denote inter-quartile range, bar denotes median, and whiskers denote range. **P* < 0.05, ***P* < 0.01, ****P* < 0.001.

SUPPLEMENTARY FIGURE 3 The proportion of probiotic, commensal, and potential pathogenic bacteria in PWH and HC. HF_ID indicated healthy individuals, F_ID indicated people with HIV.

SUPPLEMENTARY FIGURE 4 Enterotypes in the HC group. **(A)** According to the maximum Calinski–Harabasz (CH) index, the optimal number of enterotypes of the HC group were two clusters. **(B)** The principal co-ordinate analysis (PCoA) blot. **(C)** The relative abundance of the top ten species in the two enterotypes in the HC group.

SUPPLEMENTARY FIGURE 5 Taxa bar barplots of main species in ET-B, ET-P, and ET-E PWH. Each bar represents frequencies of species in gut microbiome of an individual.

SUPPLEMENTARY FIGURE 6 Alpha diversity and beta diversity between different enterotypes in HC **(A,C)** and PWH **(B,D)**. **(A,B)** Chao1 index, Shannon index, **(C,D)** Shannon index. **P* < 0.05, ***P* < 0.01, ****P* < 0.001. NS means “Not Significant” (there was no significant difference between groups).

SUPPLEMENTARY FIGURE 7 Fisher's exact probability test between low and high CD4+T-cell counts, as well as between low and high CD4/CD8 ratios.

SUPPLEMENTARY TABLE 1 Taxonomic profiles of 635 ASV in the gut microbial community of HC, which include 454 known species, 81 potentially new species, and 100 potentially higher taxa.

SUPPLEMENTARY TABLE 2 List of probiotic, commensal, and potential pathogenic bacteria detected in gut microbiota of people with HIV.

SUPPLEMENTARY TABLE 3 List of significantly different gut microbiota between the HC and the PWH groups. Red fonts indicate gut microbiota that meet the screening criteria (|log2FoldChange|>1 and FDR adjusted *P*-value < 0.05).
