# Supplementary figures and images for: Species-level taxonomic characterization of gut microbiota in HIV-infected individuals
Source: Front Microbiol. 2025 Aug 29;16:1657388. doi: 10.3389/fmicb.2025.1657388 (PMC12427029; doi:10.3389/fmicb.2025.1657388)

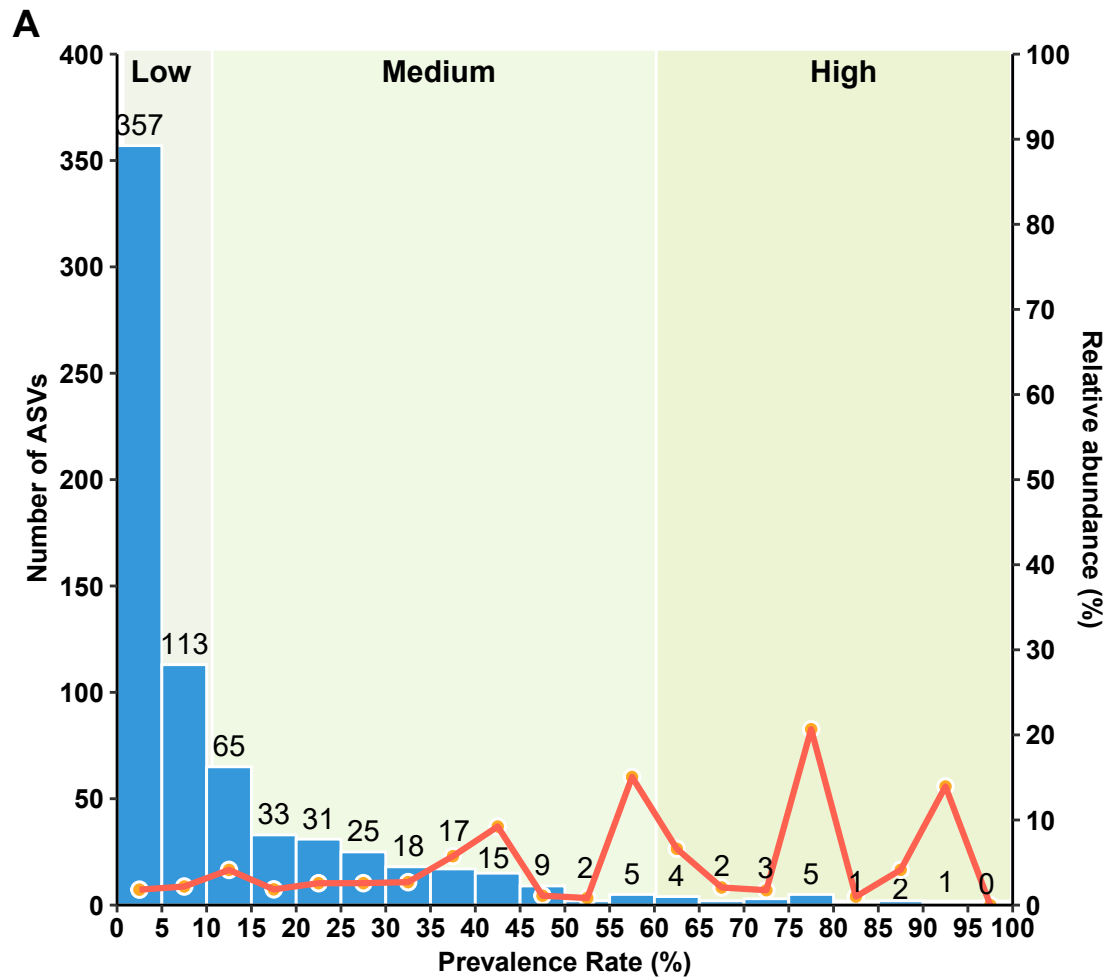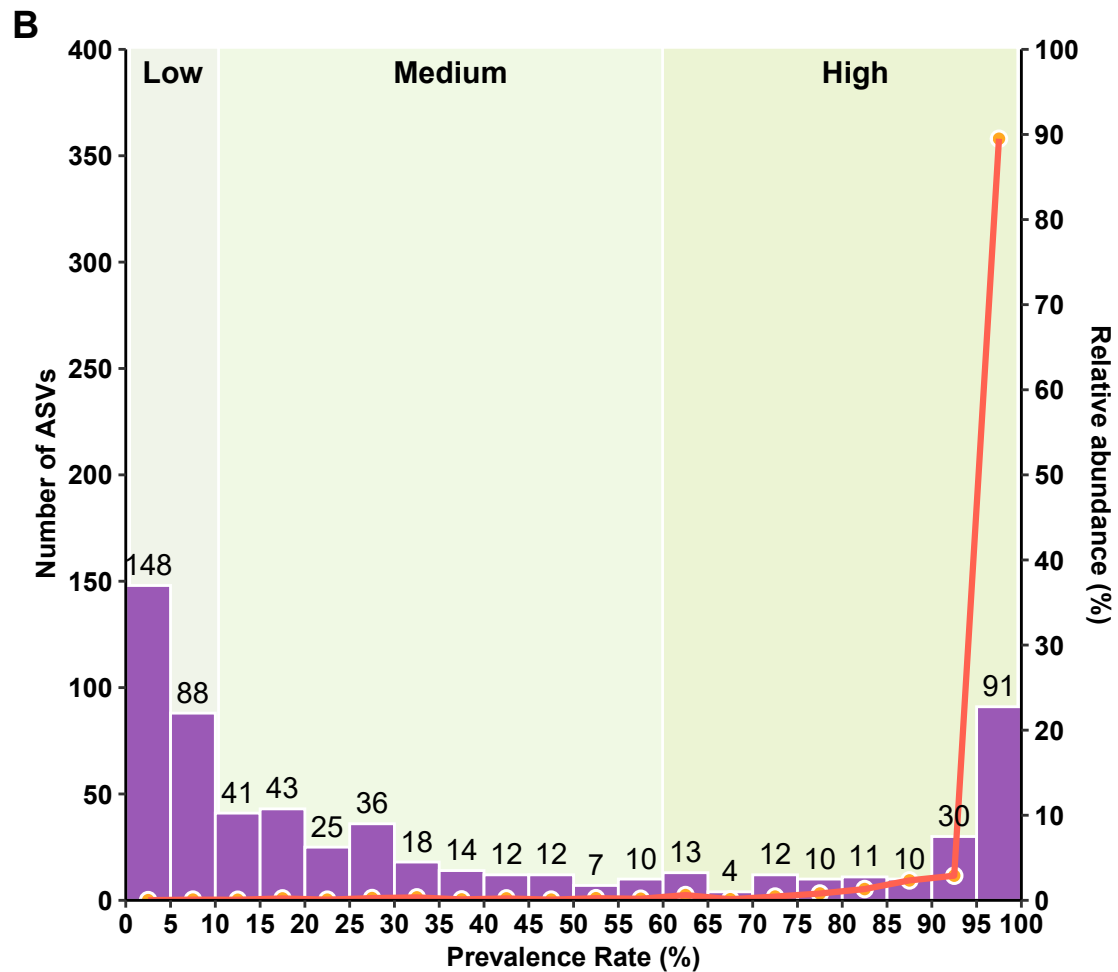

Supplement: SUPPLEMENTARY FIGURE 1 — Low (<10%), medium (10–60%), and high (>60%) prevalent bacteria groups in PWH and HC. The numbers (left axis) and the relative abundance (right axis) of the ASVs in individuals with the 5% interval. Panel (A) in the PWH group. Panel (B) in the HC group. [file Image_1.pdf]

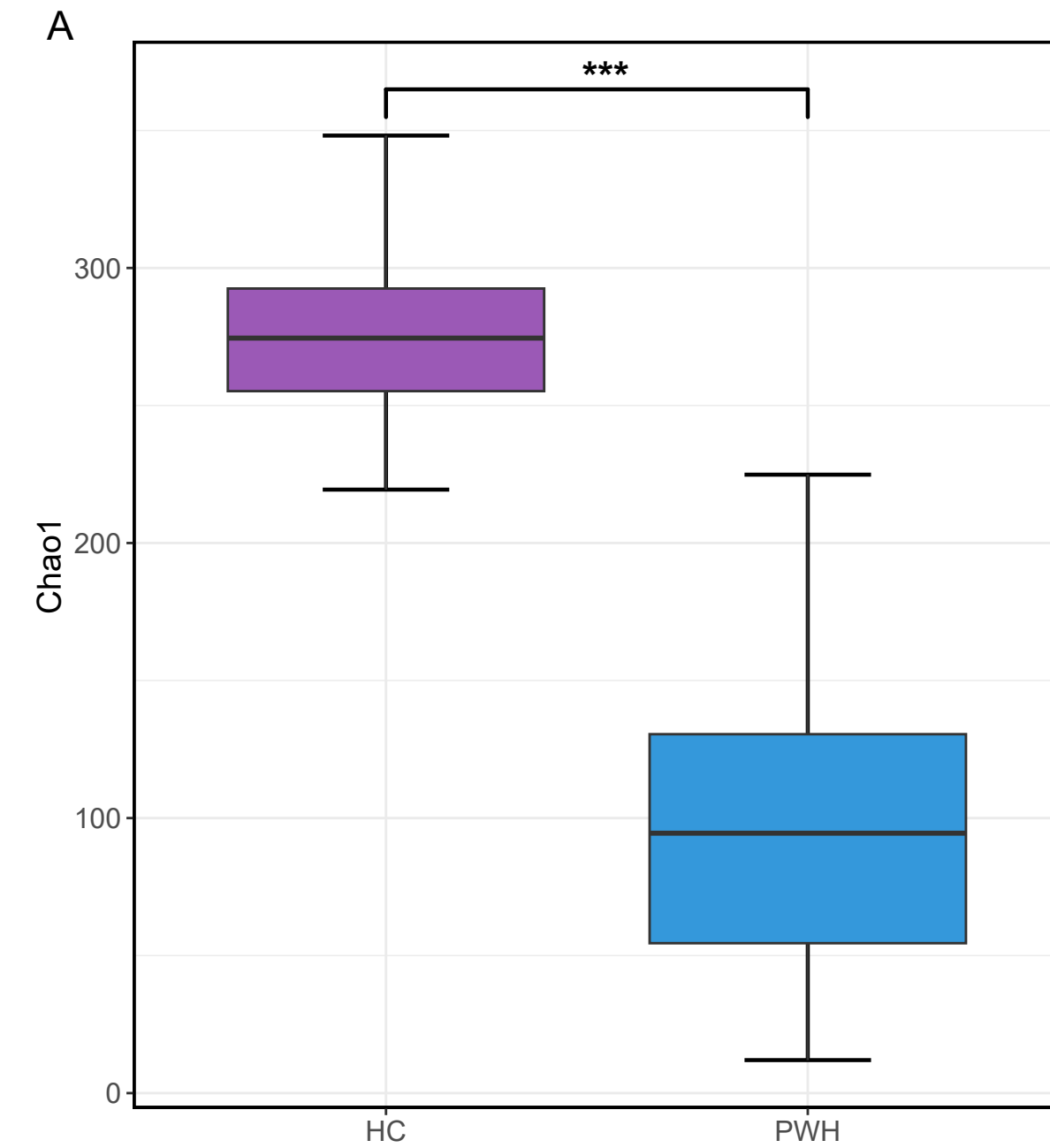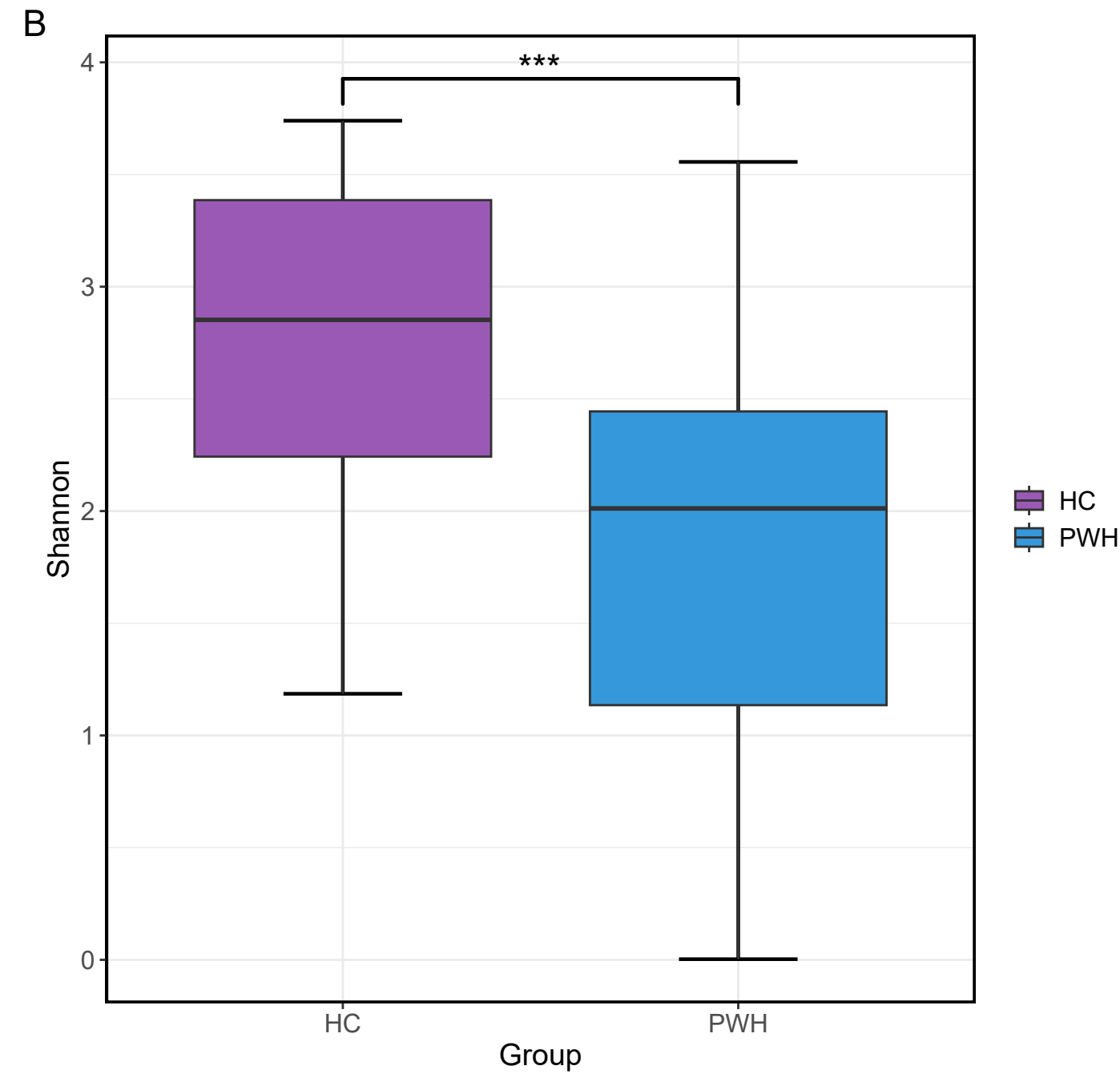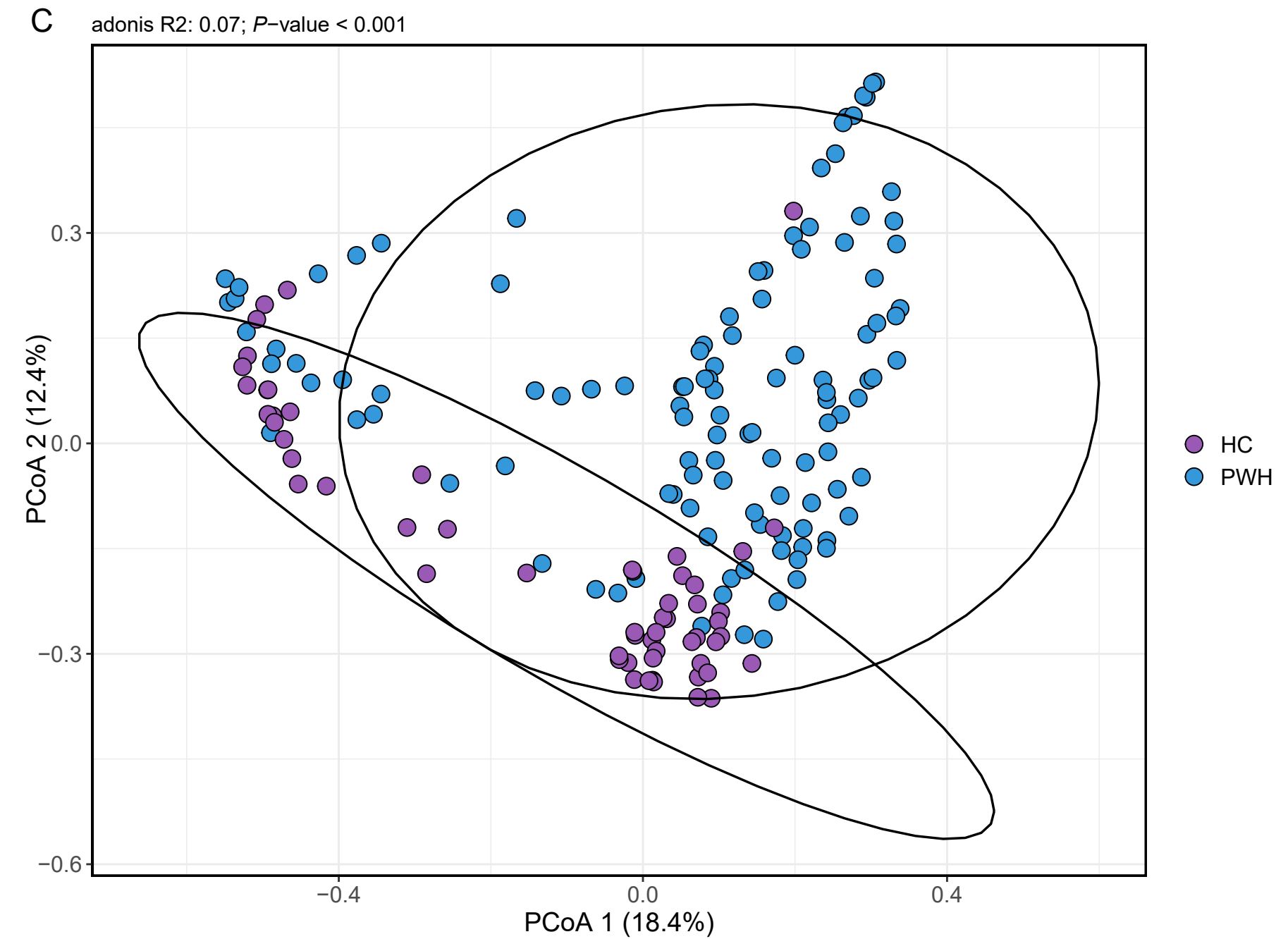

Supplement: SUPPLEMENTARY FIGURE 2 — Alpha diversity and beta diversity between PWH and HC groups. (A) Chao1 index, (B) Shannon index, (C) β diversity. P-values < 0.001. Boxes denote inter-quartile range, bar denotes median, and whiskers denote range. *P < 0.05, **P < 0.01, ***P < 0.001. [file Image_2.pdf]

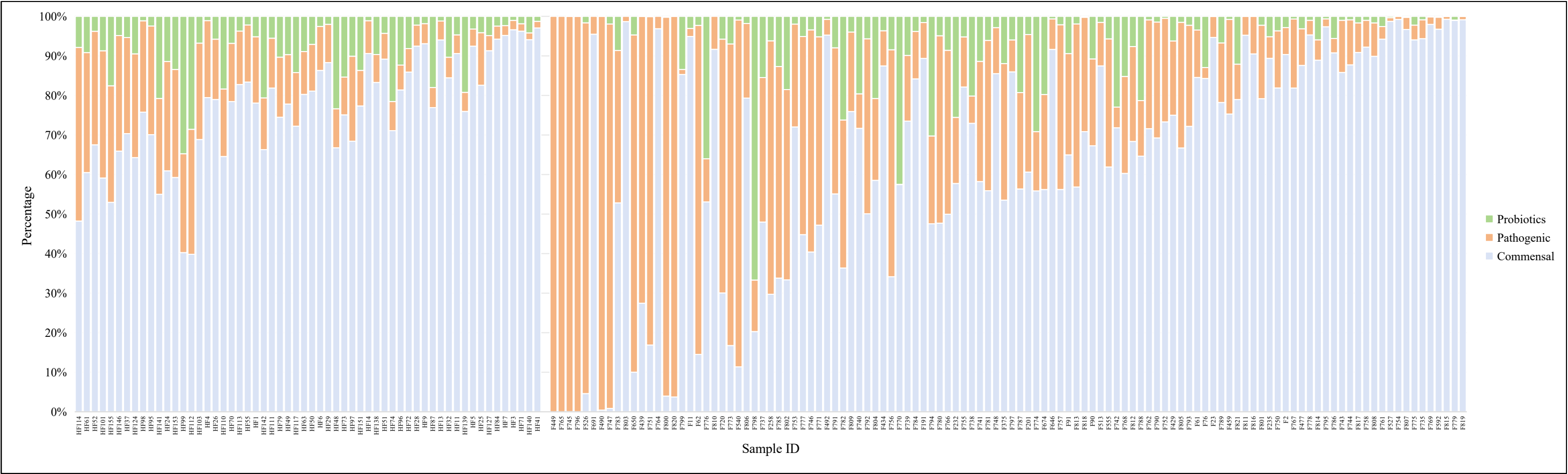

Supplement: SUPPLEMENTARY FIGURE 3 — The proportion of probiotic, commensal, and potential pathogenic bacteria in PWH and HC. HF_ID indicated healthy individuals, F_ID indicated people with HIV. [file Image_3.pdf]

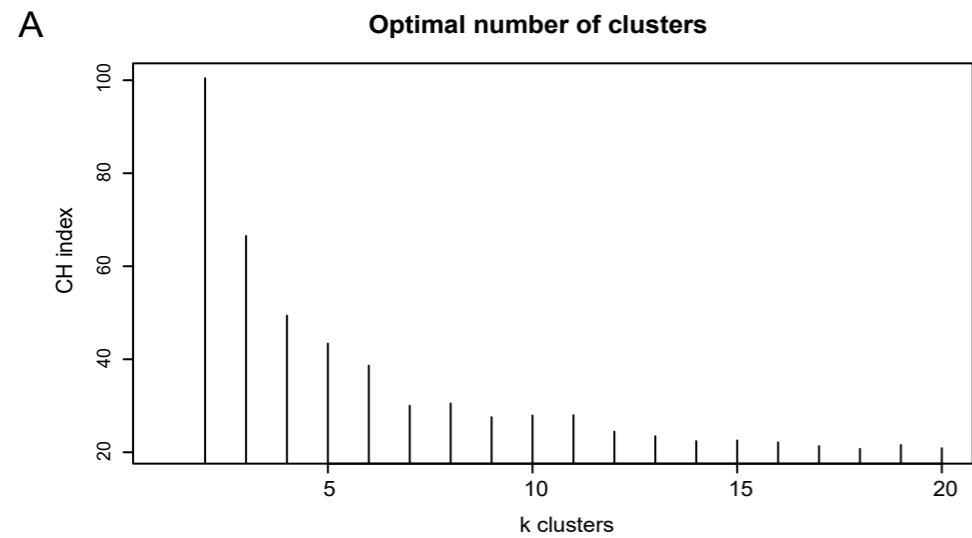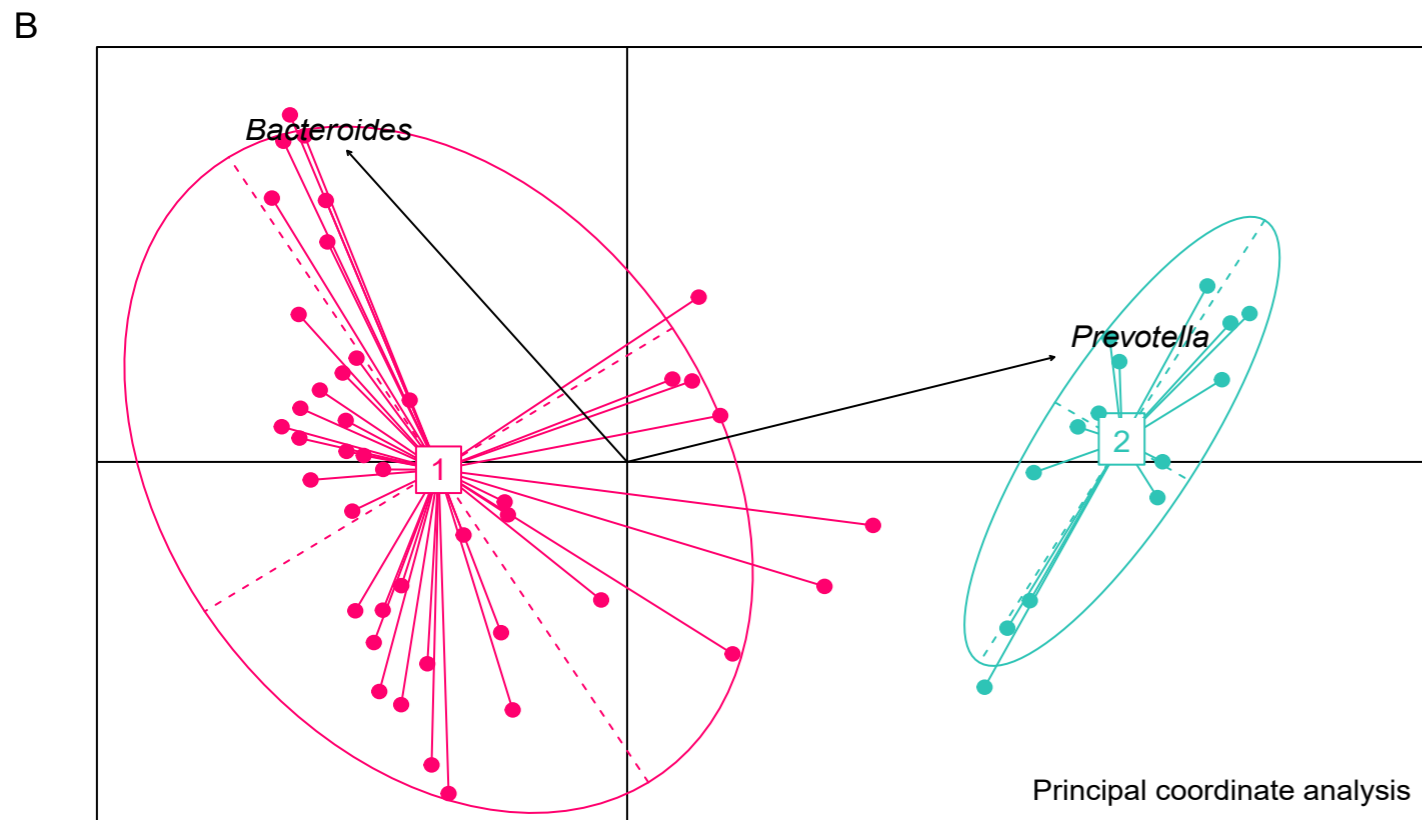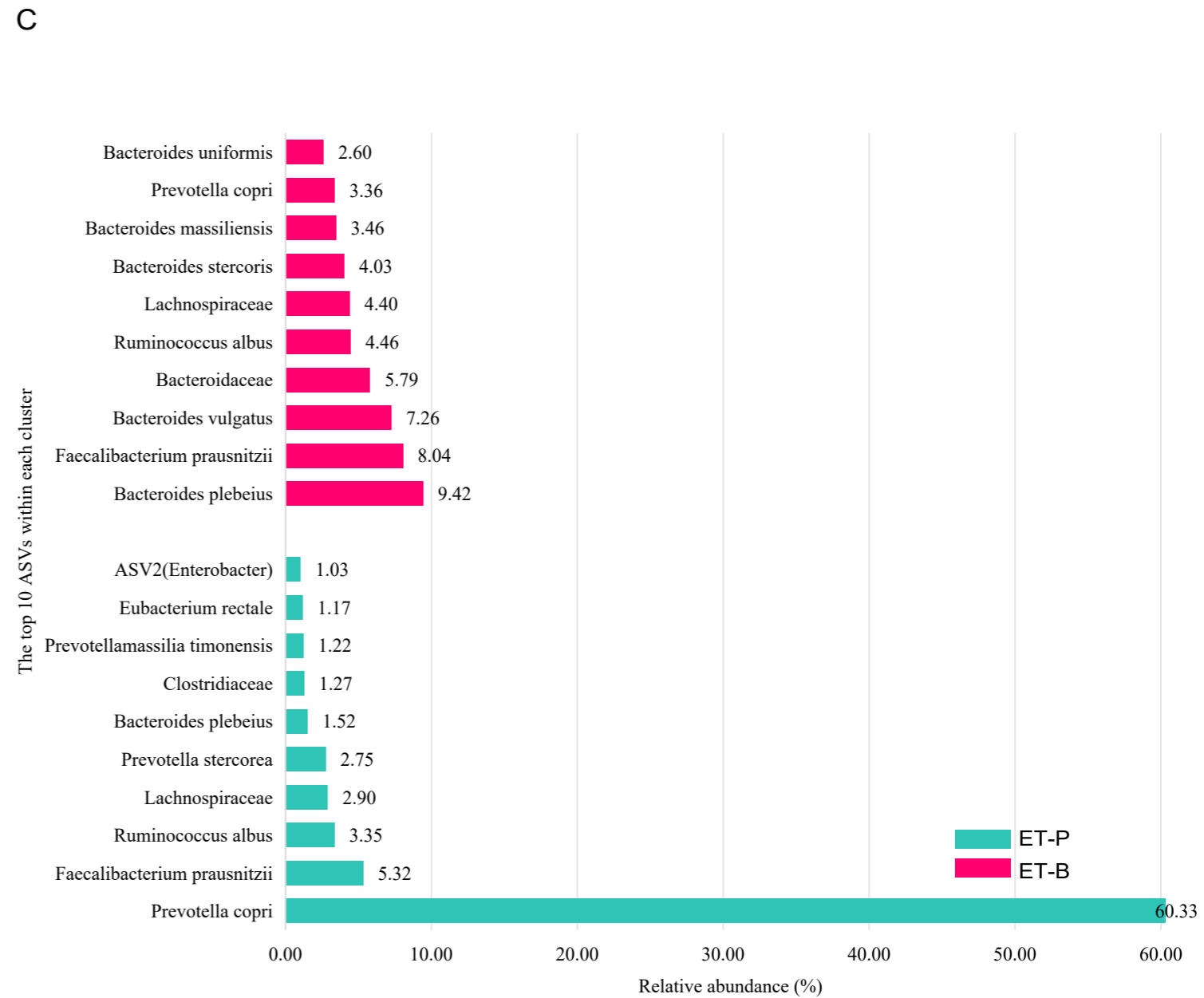

Supplement: SUPPLEMENTARY FIGURE 4 — Enterotypes in the HC group. (A) According to the maximum Calinski–Harabasz (CH) index, the optimal number of enterotypes of the HC group were two clusters. (B) The principal co-ordinate analysis (PCoA) blot. (C) The relative abundance of the top ten species in the two enterotypes in the HC group. [file Image_4.pdf]

A

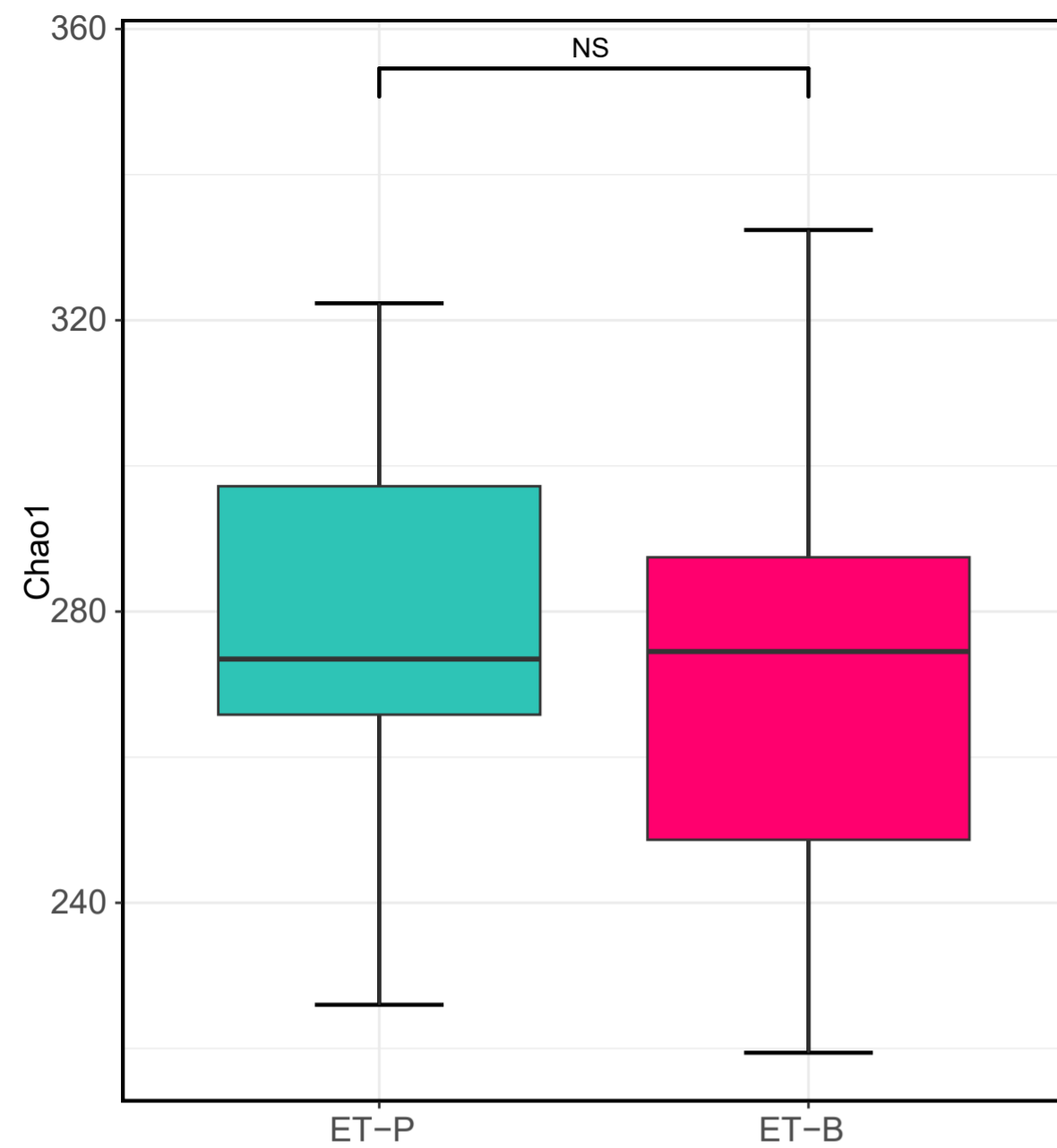

B

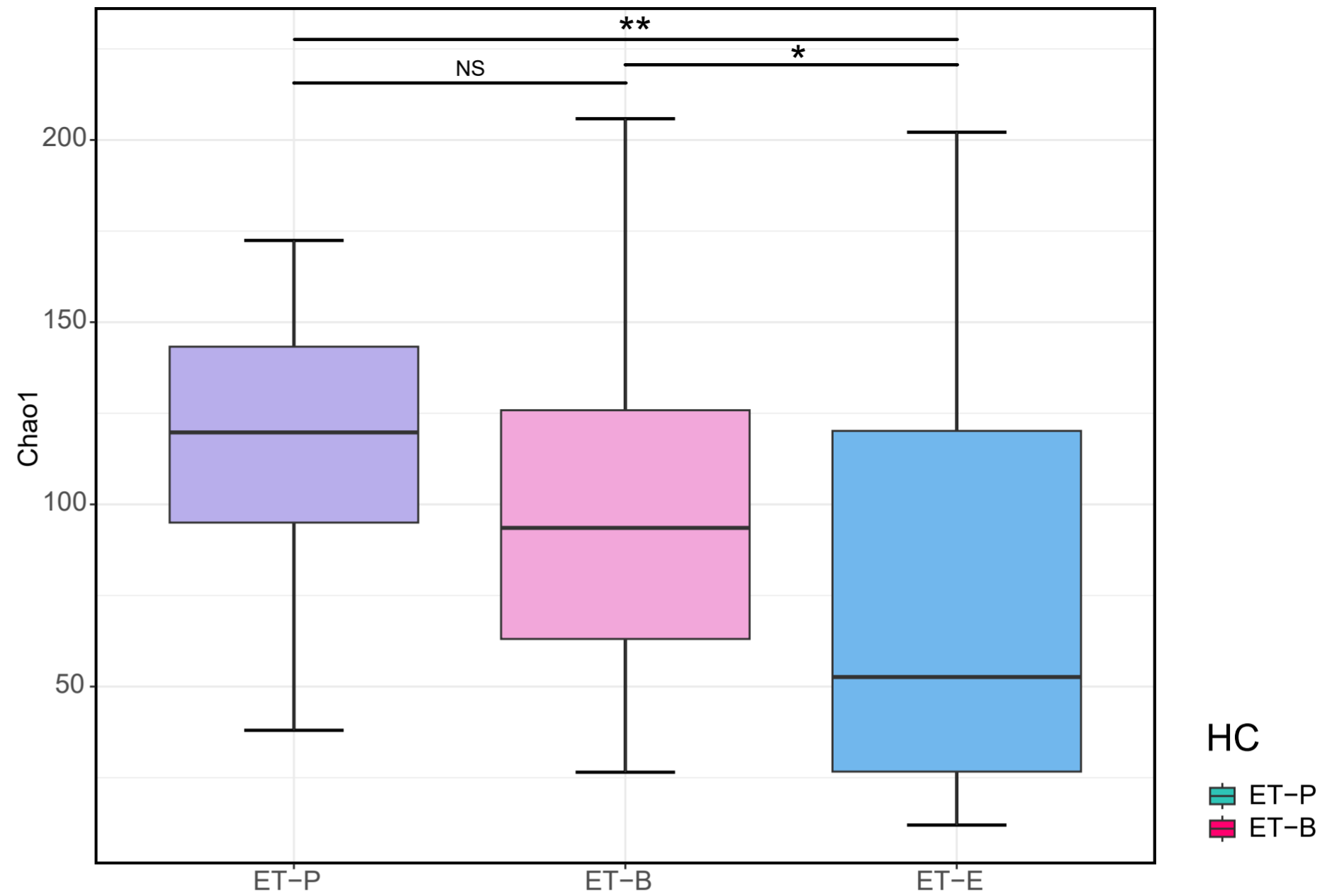

C

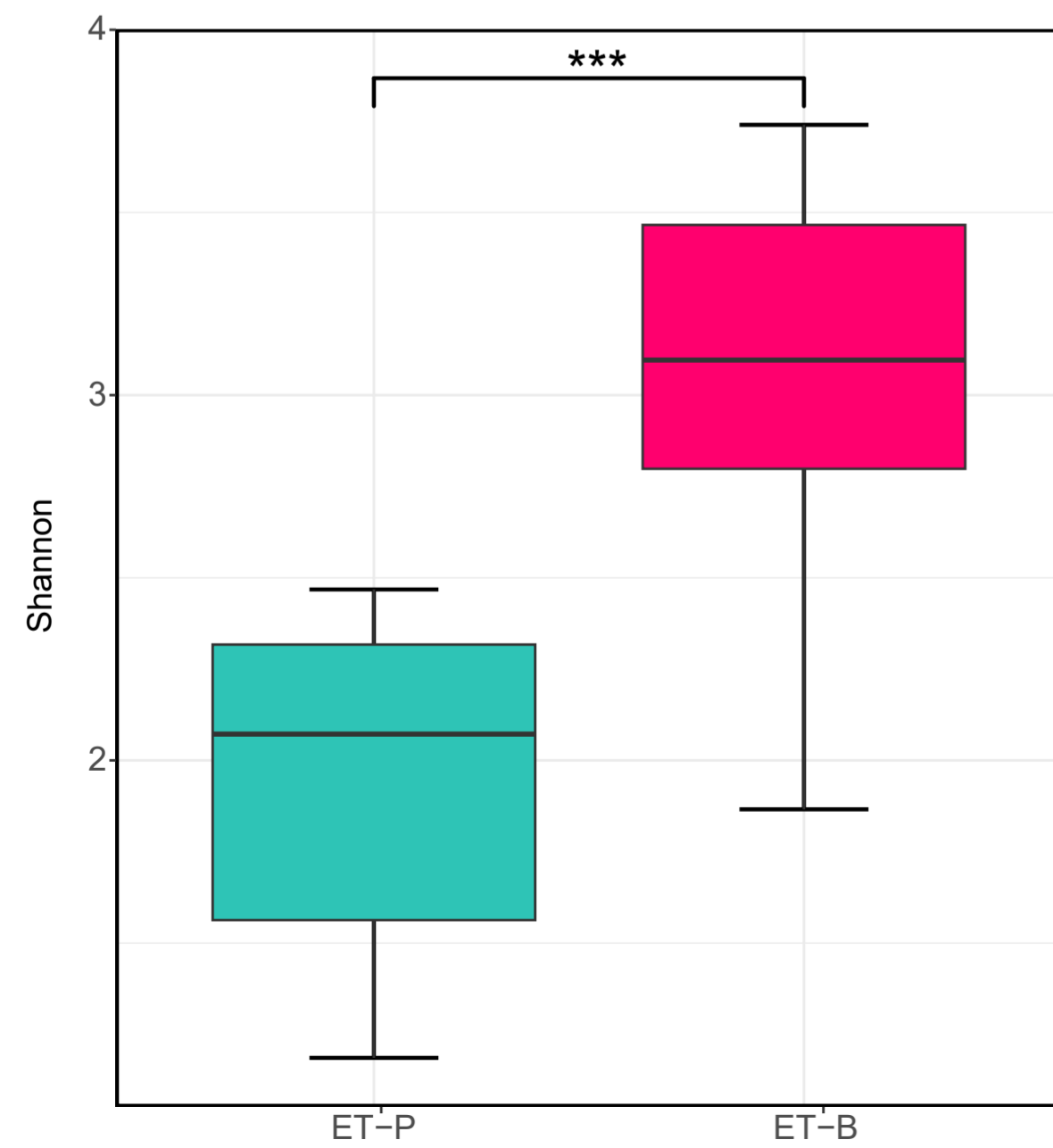

D

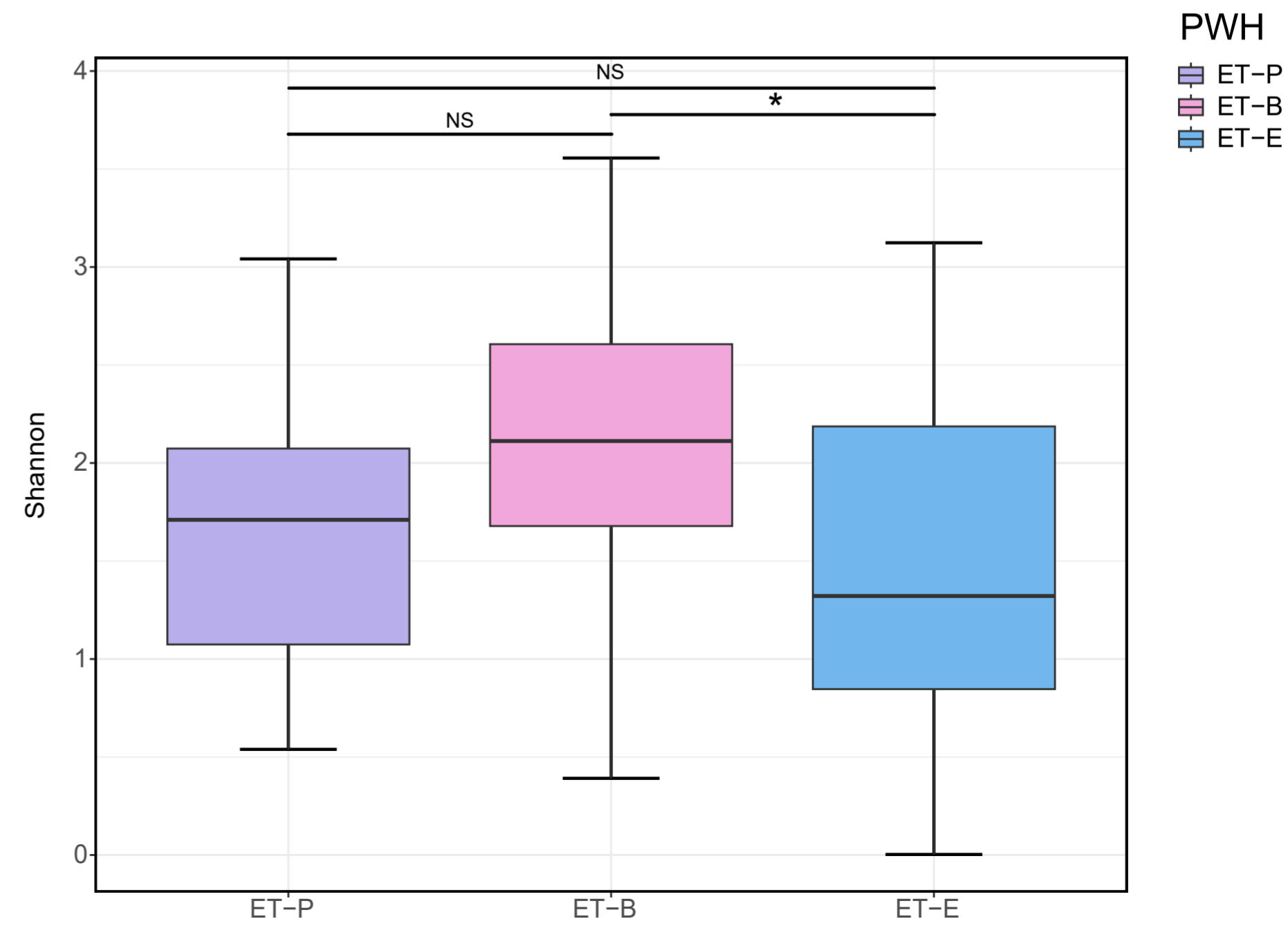

Supplement: SUPPLEMENTARY FIGURE 6 — Alpha diversity and beta diversity between different enterotypes in HC (A,C) and PWH (B,D). (A,B) Chao1 index, Shannon index, (C,D) Shannon index. *P < 0.05, **P < 0.01, ***P < 0.001. NS means “Not Significant” (there was no significant difference between groups). [file Image_6.pdf]

A

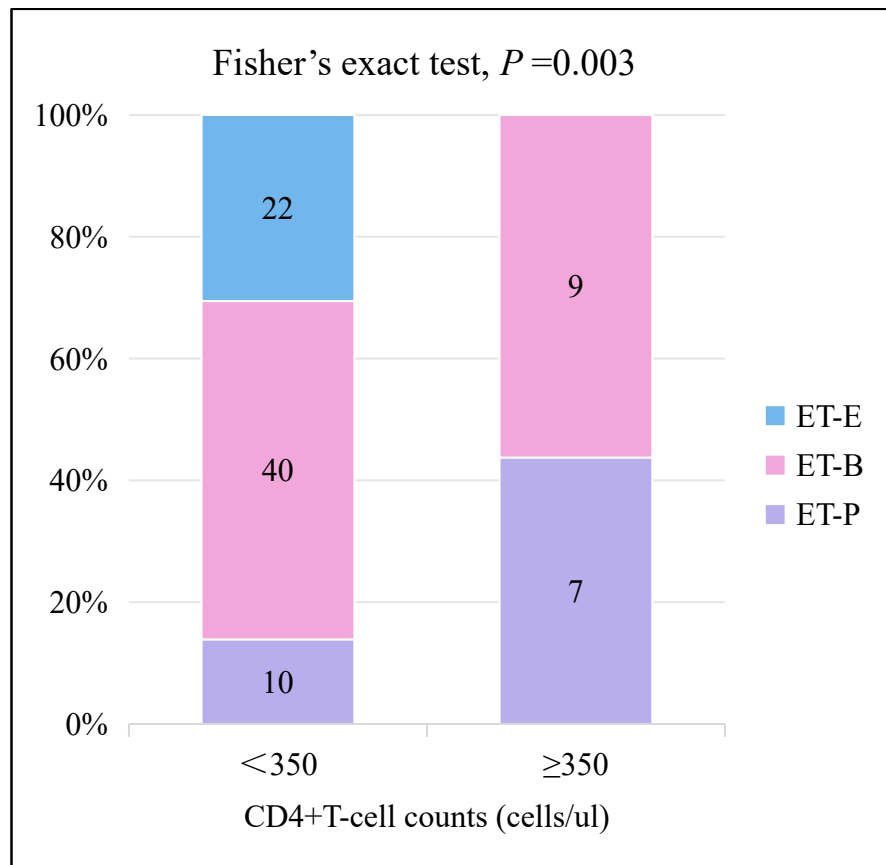

B

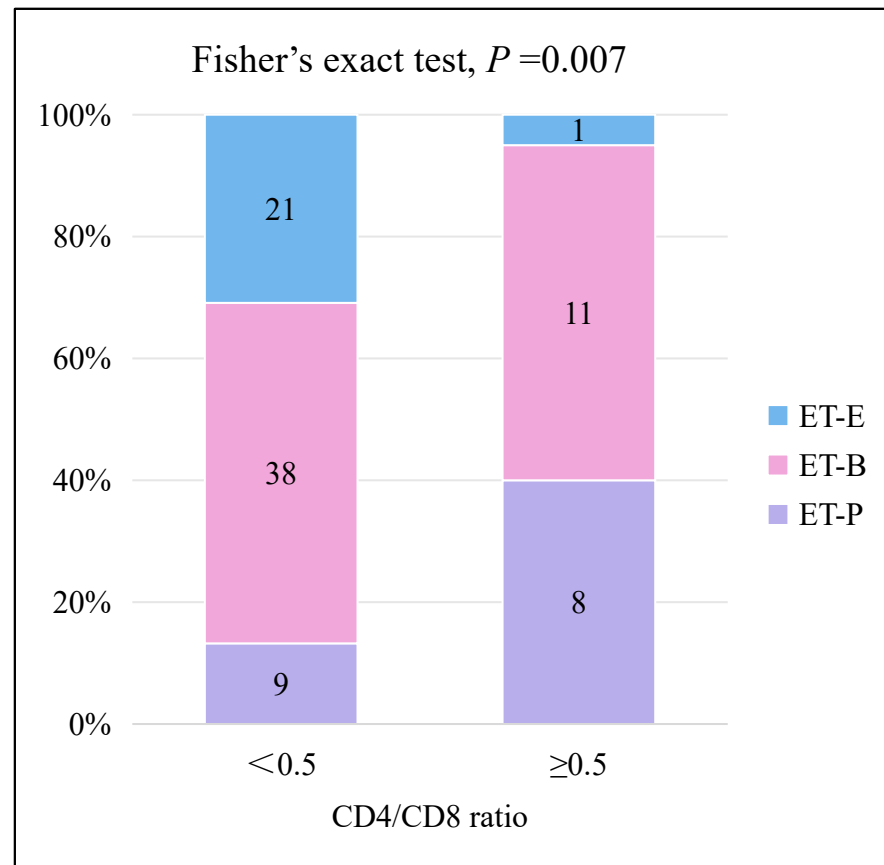

Supplement: SUPPLEMENTARY FIGURE 7 — Fisher's exact probability test between low and high CD4+T-cell counts, as well as between low and high CD4/CD8 ratios. [file Image_7.pdf]
