# Supplementary material for: Species-level taxonomic characterization of gut microbiota in HIV-infected individuals
Source: Front Microbiol. 2025 Aug 29;16:1657388. doi: 10.3389/fmicb.2025.1657388 (PMC12427029; doi:10.3389/fmicb.2025.1657388)
Supplement: SUPPLEMENTARY FIGURE 5 — Taxa bar barplots of main species in ET-B, ET-P, and ET-E PWH. Each bar represents frequencies of species in gut microbiome of an individual. [file Image_5.pdf]

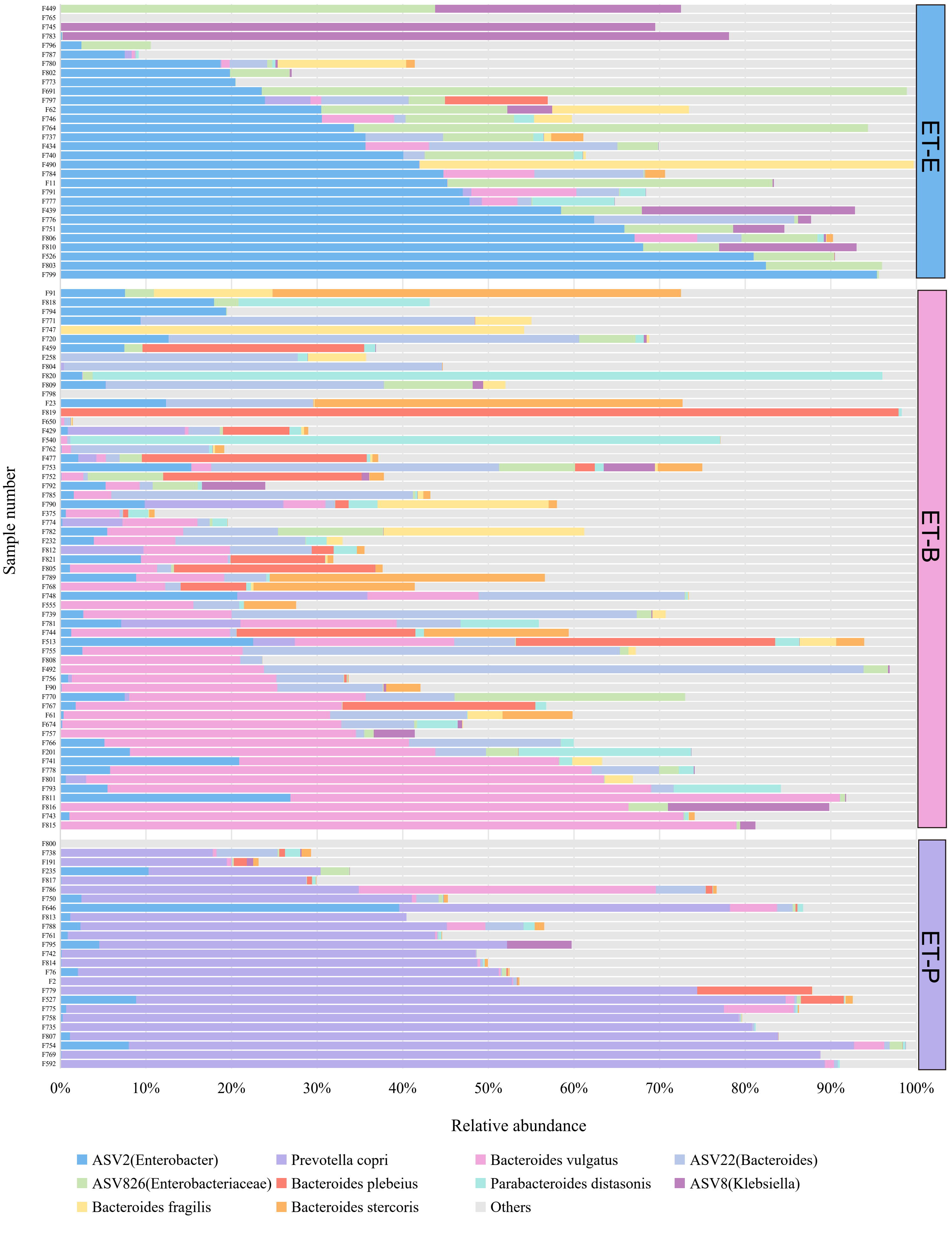
Sample number
F449
F765
F745
F783
F796
F787
F780
F802
F773
F691
F797
F62
F746
F764
F737
F434
F740
F490
F784
F11
F791
F777
F439
F776
F751
F806
F810
F526
F803
F799
ET-E
F91
F818
F794
F771
F747
F720
F459
F258
F804
F820
F809
F798
F23
F819
F650
F429
F540
F762
F477
F753
F752
F792
F785
F790
F375
F774
F782
F232
F812
F821
F805
F789
F768
F748
F555
F739
F781
F744
F513
F755
F808
F492
F756
F90
F770
F767
F61
F674
F757
F766
F201
F741
F778
F801
F793
F811
F816
F743
F815
ET-B
F800
F738
F191
F235
F817
F786
F750
F646
F813
F788
F761
F795
F742
F814
F76
F2
F779
F527
F775
F758
F735
F807
F754
F769
F592
ET-P
0%
10%
20%
30%
40%
50%
60%
70%
80%
90%
100%
Relative abundance
ASV2(Enterobacter)
Prevotella copri
Bacteroides vulgatus
ASV22(Bacteroides)
ASV826(Enterobacteriaceae)
Bacteroides plebeius
Parabacteroides distasonis
ASV8(Klebsiella)
Bacteroides fragilis
Bacteroides stercoris
Others
